# Neuroblastoma RAS viral oncogene homolog (N-RAS) deficiency aggravates liver injury and fibrosis

**DOI:** 10.1038/s41419-023-06029-y

**Published:** 2023-08-10

**Authors:** Kang Zheng, Fengjie Hao, Sandra Medrano-Garcia, Chaobo Chen, Feifei Guo, Laura Morán-Blanco, Sandra Rodríguez-Perales, Raúl Torres-Ruiz, María Isabel Peligros, Javier Vaquero, Rafael Bañares, Manuel Gómez del Moral, José R. Regueiro, Eduardo Martínez-Naves, Mohamed Ramadan Mohamed, Rocío Gallego-Durán, Douglas Maya, Javier Ampuero, Manuel Romero-Gómez, Albert Gilbert-Ramos, Sergi Guixé-Muntet, Anabel Fernández-Iglesias, Jordi Gracia-Sancho, Mar Coll, Isabel Graupera, Pere Ginès, Andreea Ciudin, Jesús Rivera-Esteban, Juan M. Pericàs, María Dolores Frutos, Bruno Ramos Molina, José María Herranz, Matías A. Ávila, Yulia A. Nevzorova, Edgar Fernández-Malavé, Francisco Javier Cubero

**Affiliations:** 1 Department of Immunology, Ophthalmology & ENT, Complutense University School of Medicine, Madrid, Spain; 2 12 de Octubre Health Research Institute (imas12), Madrid, Spain; 3 Department of Anesthesiology, Nanjing Pukou District Hospital of Chinese Medicine Central Laboratory affiliated to Nanjing University of Chinese Medicine, Nanjing, China; 4 Department of General Surgery, Ruijin Hospital, Shanghai Jiao Tong University, Shanghai, China; 5 Department of General Surgery, Wuxi Xishan People’s Hospital, Wuxi, China; 6 Department of General Surgery, the Affiliated Drum Tower Hospital of Nanjing University Medical School, Nanjing, China; 7 Department of Obstetrics and Gynaecology, Nanjing Drum Tower Hospital, The Affiliated Hospital of Nanjing University Medical School, Nanjing, China; 8 Molecular Cytogenetics and Genome Editing Unit, Human Cancer Genetics Program, Centro Nacional de Investigaciones Oncológicas (CNIO), Madrid, Spain; 9 Servicio de Anatomía Patológica Hospital General Universitario Gregorio Marañón, Madrid, Spain; 10 Servicio de Aparato Digestivo, Hospital General Universitario Gregorio Marañón, Madrid, Spain; 11 Instituto de Investigación Sanitaria Gregorio Marañón (IiSGM), Madrid, Spain; 12 Centro de Investigación Biomédica en Red de Enfermedades Hepáticas y Digestivas (CIBEREHD), Madrid, Spain; 13 Department of Cell Biology, Complutense University School of Medicine, Madrid, Spain; 14 Department of Clinical Medicine III, University Hospital Aachen, UKA, Aachen, Germany; 15 Instituto de Biomedicina de Sevilla/Hospital Universitario Virgen del Rocío/Universidad de Sevilla, Sevilla, Spain; 16 Liver Vascular Biology, IDIBAPS Biomedical Research Institute, Barcelona, Spain; 17 Department of Visceral Surgery and Medicine, Inselspital, Bern University Hospital, University of Bern, Bern, Switzerland; 18 Laboratorio de Plasticidad de Células Hepáticas y Reparación de Tejidos, Institut d´Investigacions Biomèdiques August Pi i Sunyer (IDIBAPS), Barcelona, Spain; 19 Liver Unit, Hospital Clinic, Barcelona, Spain; 20 Endocrinology Department, Vall d’Hebron University Hospital, Vall d’Hebron Institute for Research (VHIR), Barcelona, Spain; 21 Liver Unit, Internal Medicine Department, Vall d’Hebron University Hospital, Vall d’Hebron Institute for Research (VHIR), Barcelona, Spain; 22 Department of General and Digestive System Surgery, Virgen de la Arrixaca University Hospital, Murcia, Spain; 23 Laboratorio de Obesidad y Metabolismo, Instituto de Investigación Biomédica de Murcia (IMIB-Arrixaca), Murcia, Spain; 24 Hepatology Programme, Centre for Applied Medical Research (CIMA), University of Navarra, Pamplona, Spain; 25 IdiSNA, Navarra Institute for Health Research, Pamplona, Spain

**Keywords:** Homeostasis, Pathogenesis

## Abstract

Progressive hepatic damage and fibrosis are major features of chronic liver diseases of different etiology, yet the underlying molecular mechanisms remain to be fully defined. N-RAS, a member of the RAS family of small guanine nucleotide-binding proteins also encompassing the highly homologous H-RAS and K-RAS isoforms, was previously reported to modulate cell death and renal fibrosis; however, its role in liver damage and fibrogenesis remains unknown. Here, we approached this question by using N-RAS deficient (N-RAS^−/−^) mice and two experimental models of liver injury and fibrosis, namely carbon tetrachloride (CCl_4_) intoxication and bile duct ligation (BDL). In wild-type (N-RAS^+/+^) mice both hepatotoxic procedures augmented N-RAS expression in the liver. Compared to N-RAS^+/+^ counterparts, N-RAS^−/−^ mice subjected to either CCl_4_ or BDL showed exacerbated liver injury and fibrosis, which was associated with enhanced hepatic stellate cell (HSC) activation and leukocyte infiltration in the damaged liver. At the molecular level, after CCl_4_ or BDL, N-RAS^−/−^ livers exhibited augmented expression of necroptotic death markers along with JNK1/2 hyperactivation. In line with this, N-RAS ablation in a human hepatocytic cell line resulted in enhanced activation of JNK and necroptosis mediators in response to cell death stimuli. Of note, loss of hepatic N-RAS expression was characteristic of chronic liver disease patients with fibrosis. Collectively, our study unveils a novel role for N-RAS as a negative controller of the progression of liver injury and fibrogenesis, by critically downregulating signaling pathways leading to hepatocyte necroptosis. Furthermore, it suggests that N-RAS may be of potential clinical value as prognostic biomarker of progressive fibrotic liver damage, or as a novel therapeutic target for the treatment of chronic liver disease.

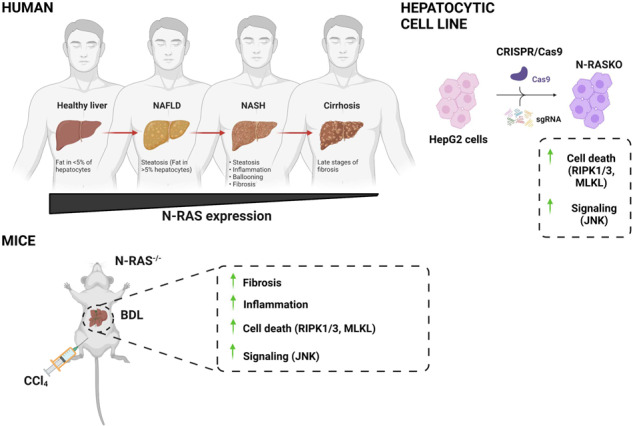

## Introduction

The small guanine nucleotide-binding proteins of the RAS family [1], encompassing in mammals the highly homologous H-RAS, N-RAS, and K-RAS (4A and 4B splice variants) isoforms, act as molecular switches to convey extracellularly derived signals into the cell interior. RAS proteins cycle between an inactive GDP-bound state and an active GTP-bound state, because of the concerted action of guanine nucleotide exchange factors (GEFs) and GTPase-activating proteins (GAPs). RAS activation is involved in a variety of key cellular processes including cell survival and death, proliferation, differentiation, and migration [1]. Although RAS proteins are highly homologous and ubiquitously expressed, differences in their expression in various tissues, along with a distinct subcellular distribution and recruitment of downstream effectors, suggest that each RAS isoform may subsume a specialized and specific function [2–4].

Progressive liver damage, including fibrosis, is a hallmark of chronic liver diseases. Liver fibrogenesis is a dynamic process characterized by scar healing and extracellular matrix (ECM) accumulation, in response to chronic liver injury of various types of insults [5]. Advanced chronic liver disease (ACLD), sometime referred to as advanced fibrosis or cirrhosis, represents one late stage of chronic liver disease of different etiology, including metabolic-associated fatty liver disease, alcohol, viral infections, and autoimmune liver disease [6]. Liver biopsy remains as the gold standard method for the diagnosis of ACLD, by determining the grade of steatosis, necroinflammation and fibrosis simultaneously [6]. Although our current understanding of liver fibrosis improved in the past decades, the underlying molecular mechanisms are still poorly defined and most treatments have proved ineffective. Thus, approved medications for ACLD-related fibrosis are still lacking.

Signaling from Ras proteins has been implicated in fibrogenesis. Farnesylthiosalicylic acid (FTS), a pan-RAS antagonist, reduced fibrosis in experimentally-induced liver cirrhosis [7], although the specific Ras isoforms involved were not identified. N-RAS, in particular, was previously reported to modulate renal fibrosis [8], but its involvement in liver fibrogenesis has remained unknown. Hence, we sought to investigate the role of N-RAS in fibrotic liver disease by using mice deficient for N-RAS and well-established experimental hepatotoxic models (carbon tetrachloride, CCl_4_ and bile duct ligation, BDL) [9, 10]; in combination with analyses of N-RAS expression in chronic liver disease patients with distinct degree of fibrosis.

## Results

### N-RAS deficiency exacerbates CCl_4_-induced liver fibrosis and immune cell infiltration

We sought to determine whether N-RAS plays a key function in the induction and development of liver injury and fibrosis by analyzing gene-targeted mice specifically lacking N-RAS (Suppl. Figure 1A).

Chronic administration of carbon tetrachloride (CCl_4_), a widely accepted model of advanced chronic liver disease (ACLD) [11], induced N-RAS gene expression in wild-type mice (Suppl. Figure 1B). Since RAS family is comprised by two other genes, namely H-RAS and K-RAS, we also checked mRNA expression of these isoforms. Our result indicated that both H-RAS and K-RAS increased significantly in N-RAS^−/−^ compared with N-RAS^+/+^ mice, after 28 days of CCl_4_ treatment (Suppl. Figure 1C, D).

**Fig. 1:**
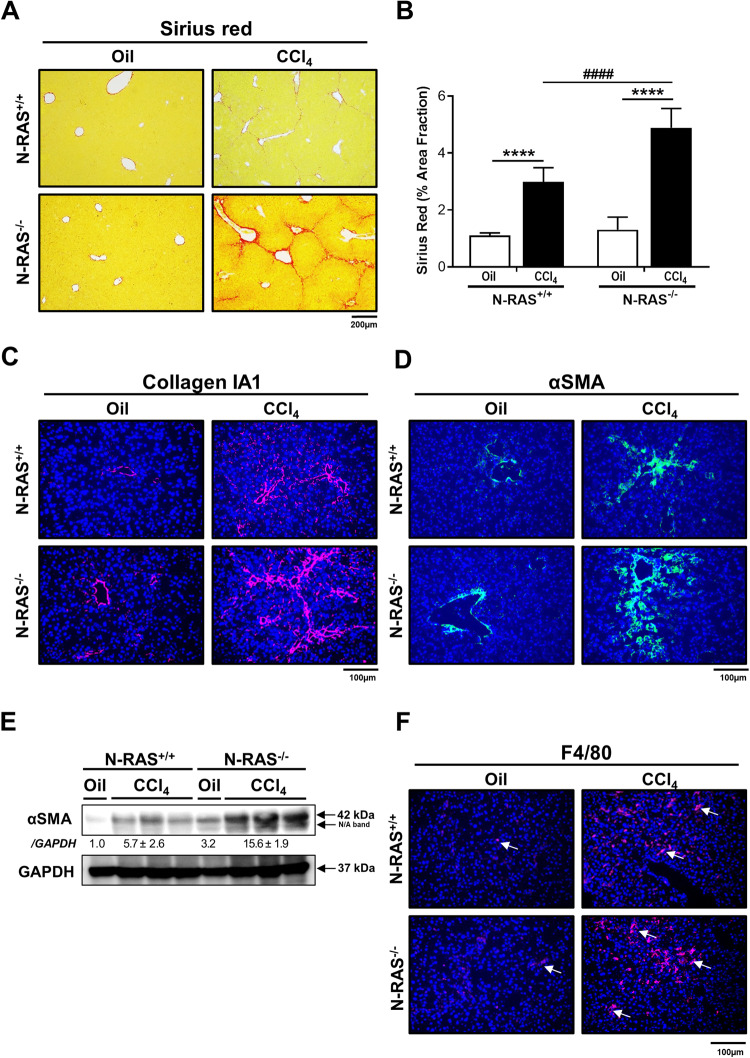
N-RAS deficiency exacerbates CCl_4_-induced liver fibrosis and immune cell infiltration. Representative Sirius red (SR) staining (**A**) and (**B**) quantification of SR areas in paraffin sections from N-RAS^+/+^ and N-RAS^−/−^ livers, after 28 days CCl_4_ treatment. Immunofluorescence staining for Collagen IA1 (**C**) and (**D**) α-SMA (**D**) was performed in liver cryosections from N-RAS^+/+^ and N-RAS^−/−^ animals. **E** Liver extracts were prepared and analyzed by immunoblot using α-SMA. GAPDH was used as housekeeping protein. **F** Representative hepatic F4/80 immunofluorescence staining from N-RAS^+/+^ and N-RAS^−/−^ mice, after 28 days CCl_4_ treatment. Arrows (→) indicate positive cells. Values represent mean ± SEM from 6–8 mice per group (intragroup ****p < 0.0001; intragroup ^####^*p* < 0.0001).

Thus, we challenged N-RAS^+/+^ and N-RAS^−/−^ animals with CCl_4_ for a period of 28 days (Fig. 1). Previous reports identified N-RAS as an important modulator of fibroblast function and fibrosis in the kidney [8]. In the liver, treatment with CCl_4_ significantly induced higher quantitative Sirius Red (SR) staining in livers from N-RAS^*−/−*^ compared with N-RAS^+/+^ animals (Fig. 1A, B). The moderate to severe fibrosis observed by SR was confirmed by immunofluorescence (IF) and mRNA expression of collagen IA1 and alpha-smooth muscle actin (αSMA), a marker of hepatic stellate cell (HSC) activation (Fig. 1C, D, Suppl. Figure 2A, B). These findings were validated by increased αSMA protein expression in liver tissue of N-RAS^−/−^ compared with N-RAS^+/+^ mice (Fig. 1E).

We next evaluated immune cell infiltration in response to liver injury, a feature of the pathophysiology of chemically-induced liver fibrosis. We analyzed the presence of CD45^+^ (leukocytes; Suppl. Figure 3A, B), CD11b^+^ (mostly myeloid lineages; Suppl. Figure 3C, D), and F4/80^+^ (macrophages; Fig. 1F, Suppl. Figure 3E) immune cells by IF in liver sections of corn-oil and CCl_4_-treated N-RAS^+/+^ and N-RAS^−/−^ mice. Our data indicated significantly increased abundance of the immune cells expressing the above surface markers in the livers of CCl_4_-treated N-RAS^−/−^ mice compared to N-RAS^+/+^ counterparts. In addition, flow cytometric analyses of liver leukocytes (Suppl. Figure 4) revealed significantly increased frequencies of αβ T lymphocytes, including CD8^+^ cells (Suppl. Figure 4A, B), and B lymphocytes (Suppl. Figure 4F), but reduced CD4^+^, NKT and γδ T cells (Suppl. Figure 4C–E) in oil-treated N-RAS^−/−^ compared to N-RAS^+/+^ mice. In CCl_4_-treated animals, the relative abundance of αβ T and B cells were increased only in N-RAS^−/−^ mice (Suppl. Figure 4A, F); although proportions of CD8^+^ and CD4^+^ subsets within the αβ T cell compartment were comparable in both types of mice (Suppl. Figure 4B, C). Further, a tendency towards a higher percentage of NKT and γδ T cells was observed in CCl_4_-treated N-RAS^−/−^ compared to N-RAS^+/+^ mice (Suppl. Figure 4D, E). Overall, these results indicate that N-RAS deficiency is associated with exacerbation of liver fibrosis and increased infiltration of both myeloid and lymphoid immune cells after chemically-induced liver damage.

### N-RAS deficiency is associated with aggravated liver injury, cell death, and increased compensatory proliferation after chronic CCl_4_ challenge

**Fig. 2:**
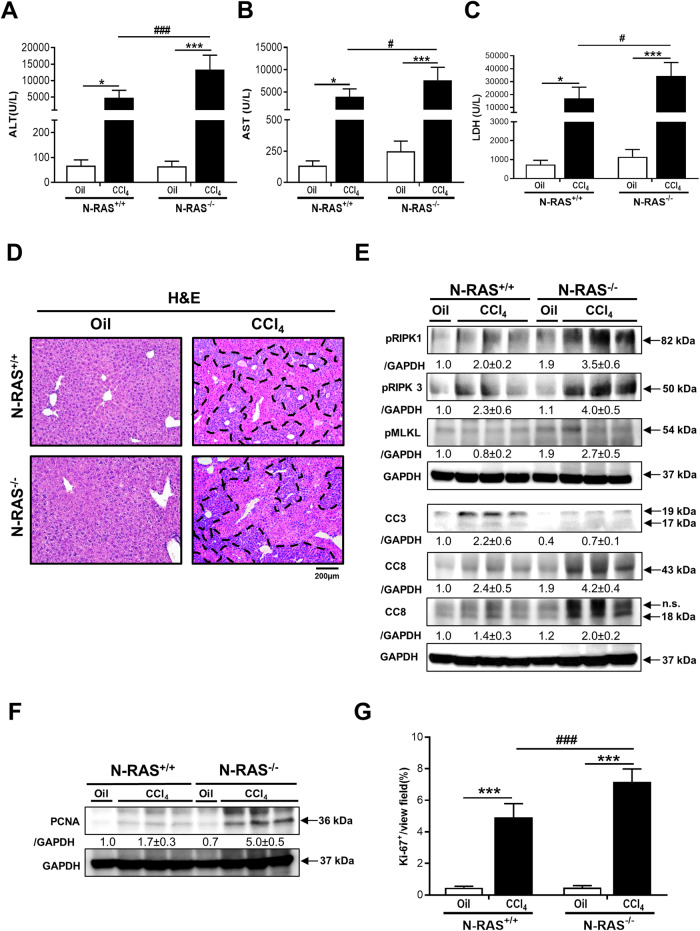
N-RAS deficiency is associated with aggravated liver injury, cell death, and increased compensatory proliferation after chronic CCl_4_ challenge. **A**–**C** Serum levels of ALT (**A**), AST (**B**), and LDH (**C**) were determined, 28 days after CCl_4_ treatment. **D** Representative H&E staining of livers. **E** Protein analysis by Western Blot of pRIK1/3, pMLKL, CC3, CC8, and **F** PCNA. GAPDH was used as housekeeping protein. **G** Immunofluorescence staining for Ki-67 of liver cryosections and positive cell quantification was performed, 28 days after CCl_4_ treatment. Values represent mean ± SEM (*N* = 6–8, intragroup, *p < 0.05–***p < 0.001; intergroup ^#^*p* < 0.05–^###^*p* < 0.001). Dotted area indicates necrotic foci.

Next, we sought to evaluate liver damage by analyzing serum biochemical markers. Interestingly, ALT, AST, and LDH were significantly increased in N-RAS^−/−^ compared with N-RAS^+/+^ animals (Fig. 2A–C), indicating that N-RAS deficiency increased chemically-induced liver injury. Macroscopically, explanted livers showed significantly increased liver weight (LW) *versus* body weight (BW) ratio in N-RAS^−/−^ compared with N-RAS^+/+^ animals (Suppl. Figure 5A, B). Blinded histopathological examination of liver samples by an expert pathologist revealed increased hepatocyte ballooning and apoptotic bodies, extensive necrotic periportal areas, and a tendency towards increased biliary foci (Fig. 2D, Suppl. Figure 5C) when N-RAS^−/−^ mice were compared to N-RAS.

CCl_4_ is metabolized by cytochrome P450 2E1 (CYP2E1) to produce the highly reactive trichloromethylperoxy radical (CCl_3_OO^●^) which initiates lipid peroxidation. After propagation of the peroxidation process, lipids are finally degraded in small molecules such as 4-hydroxynonenal (HNE), which are highly reactive aldehydes that can form protein and DNA adducts [12]. Interestingly, compared to the wild-type mice, increased 4-HNE accumulation was found in the liver of N-RAS-deficient mice, after CCl_4_ administration (Suppl. Figure 5D, E).

Cell death and compensatory proliferation represent a basic biological response defining the outcome in most hepatic disease conditions [13]. To further understand the mechanisms potentially regulated by N-RAS, we studied the levels of proteins related to cell death after chronic CCl_4_ treatment. Phosphorylated RIPK1/3, pMLKL, and cleaved caspase-8 (CC8) were overexpressed in N-RAS^−/−^ livers, whereas cleaved caspase-3 (CC3) was under expressed in these animals, compared with N-RAS^+/+^ mice (Fig. 2E). Thus, while necroptosis was characteristic of N-RAS*-*deficient livers, apoptosis was the major mode of cell death in livers from N-RAS^+/+^ mice. Moreover, overexpression of hepatic PCNA was more noticeable in N-RAS^−/−^ compared with N-RAS^+/+^ (Fig. 2F, G) mice. Altogether, these results indicated that N-RAS-deficient mice show aggravated liver injury, lipid peroxidation, necroptotic cell death, and higher compensatory proliferation upon CCl_4_-induced liver injury.

### N-RAS deficiency aggravates BDL-induced liver injury and fibrosis

**Fig. 3:**
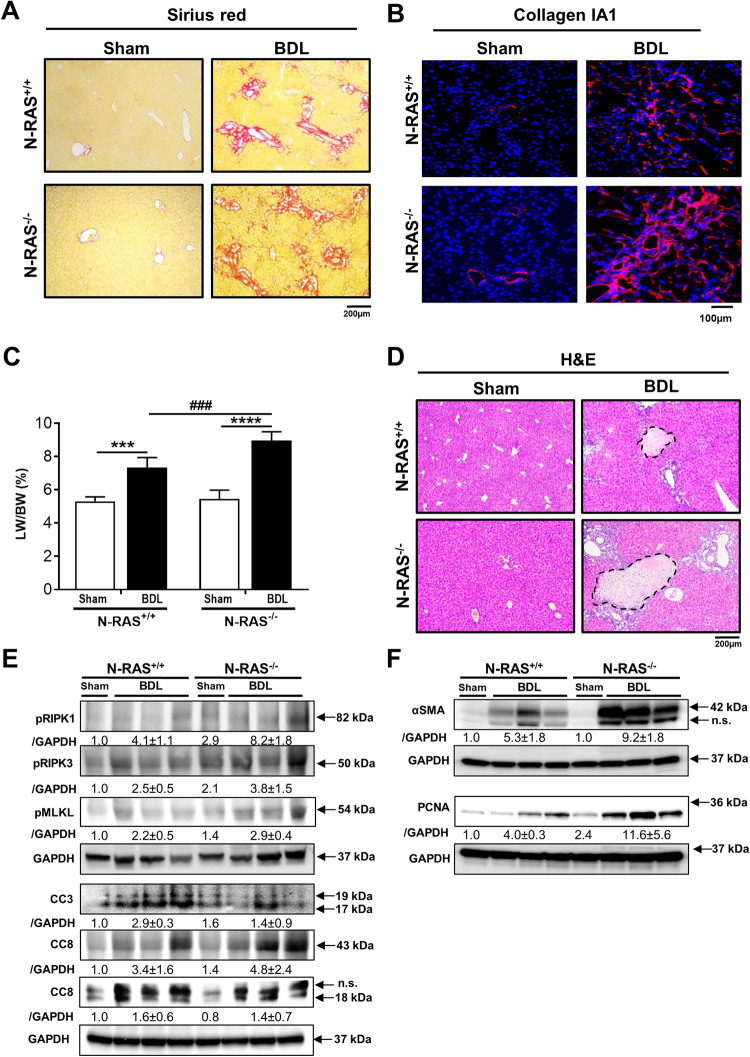
N-RAS deficiency aggravates BDL-induced liver injury and fibrosis. **A** Representative Sirius red (SR) staining (**A**) was performed in paraffin sections of N-RAS^+/+^ and N-RAS^−/−^ livers, 28 days after CCl_4_. **B** Immunofluorescence for Collagen IA was carried out in the cryosections of the same animals. **C** Liver weight (LW) versus body weight (BW) ratio was calculated and represented. **D** Representative H&E staining of *N-Ras*^*+/+*^ and *N-Ras*^*−/−*^ mice, 28 days after BDL. **E** Immunoblots were performed using pRIK1/3, pMLKL, CC3, CC8 antibodies. GAPDH was used as housekeeping protein. **F** Protein expression of α-SMA and PCNA was evaluated by Western blot. GAPDH was used as housekeeping protein. Values represent mean ± SEM from 5–6 mice per group (**p < 0.01; ***p < 0.001; ****p < 0.0001). Dotted areas indicate necrotic foci.

The obstruction of the common bile duct by BDL causes bile to accumulate in the liver, leading to hepatic injury, inflammation and, ultimately, peribiliary fibrosis and cirrhosis [11]. Given the results in the chemically-induced liver fibrosis model, BDL was employed as a second preclinical model of liver damage and fibrosis to test the impact of N-RAS deficiency. BDL for 28 days caused collagen deposition around the bile ducts, as determined by quantitative SR staining, collagen IA1 immunofluorescence, the expression of genes related to ECM and inflammation (Coll IA1, Coll III, αSMA, TIMP1, and IL1ß) and the protein levels of αSMA. All these parameters were specifically increased in N-RAS^−/−^ compared with N-RAS^+/+^ mice after BDL (Fig. 3A, B, F, Suppl. Figure 6A–F). Moreover, immunostaining and quantification of liver infiltrating cells demonstrated significantly increased CD45, CD11b, and F4/80 positive cells in the livers of N-RAS^−/−^ compared with N-RAS^+/+^ mice after BDL (Suppl. Figure 7A–F). These data indicated that the absence of N-RAS significantly increased ECM deposition and leukocyte recruitment in the injured liver.

LW/BW ratio was also significantly increased in BDL-treated N-RAS^−/−^ compared with N-RAS^+/+^ mice (Fig. 3C, Suppl. Figure 8A). Histopathological examination of N-RAS^−/−^ livers showed a more intense inflammation accompanied by alteration of the hepatic architecture and extensive necrotic foci, compared with N-RAS^+/+^ animals (Fig. 3D, Suppl. Figure 8B).

To deepen into the mechanisms associated to the increase in liver fibrosis observed in N-RAS-deficient mice subjected to BDL, we investigated oxidative stress, cell death, and compensatory proliferation. In agreement with the results using the CCl_4_ model, significantly elevated lipid peroxides (4HNE) were characteristic of N-RAS^−/−^ livers (Suppl. Figure 8C, D). Moreover, increased hepatic expression of canonical necroptosis pathways, including CC8, pRIK1/3, and pMLKL, along with CC3 under expression, was observed in N-RAS^−/−^ animals (Fig. 3E). Finally, PCNA protein levels were also significantly increased in N-RAS^−/−^ compared with N-RAS^+/+^ counterparts (Fig. 3F).

Altogether these data indicate that N-RAS deficiency exacerbates liver fibrosis, accompanied by lipid peroxidation, cell death, and compensatory proliferation in cholestatic liver injury.

### N-RAS deficiency alters the profile of gene expression induced by experimental liver injury

**Fig. 4:**
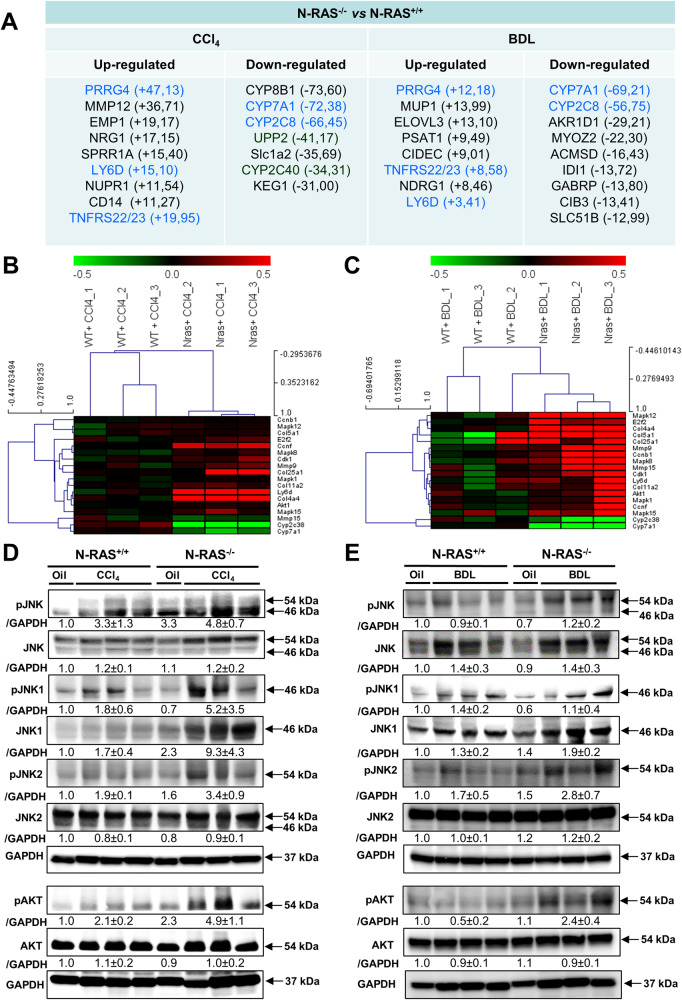
N-RAS deficiency alters the profile of gene expression induced by experimental liver injury and causes overactivation of the JNK and AKT pathways. **A** Ingenuity Pathway Analysis (IPA) was performed in N-RAS^+/+^ and N-RAS^−/−^ livers, 28 days both after CCl_4_ (left column) and BDL (right column). In blue color, common genes in both models are highlighted. Gene array analysis was performed in N-RAS^+/+^ and N-RAS^−/−^ livers, 28 days after CCl_4_ (**B**) or BDL (**C**). Correlation of the fold induction of genes in hepatocytes and liver is shown. Log2 expression values of the individual mice were divided by the mean of the sham-operated mice. Log ratios were saved in a .txt file and analyzed with the Multiple Experiment Viewer. Top up- and downregulated genes are shown (red: upregulated; green: downregulated, *n* = 3 for each model of liver fibrosis, 2.0 < FC > −2.0). **D**, **E** Immunoblotting for pJNK, JNK, pJNK1, JNK1, pJNK2, JNK2, pAKT, and AKT) was performed in liver extracts of N-RAS^+/+^ and N-RAS^−/−^ livers, 28 days after CCl_4_ (**D**) and BDL (**E**), respectively. GAPDH was used as loading control.

To gain insight into the differential signaling pathways triggered in N-RAS^−/−^ *versus* N-RAS^+/+^ mice during liver injury and fibrosis, we performed microarray analyses of livers 28 days after CCl_4_ treatment and BDL surgery, respectively (Fig. 4A–C). Interestingly, genes related to liver fibrosis and inflammation (TnfR and Ly6D) were upregulated in N-RAS knockout mice challenged either with CCl_4_ or with BDL (Fig. 4A). In turn, downregulation of genes related to biological regulation (Cyp7A1/2C8) was evident in this analysis (Fig. 4A–C). Gene clustering using a heatmap revealed significant upregulation in the expression of genes that were related to matrix deposition and collagen synthesis (Mmp9/155, Col4a4), cell cycle and proliferation (Cdk1, Ccnb1, Ccnf, and E2f2), and MAPK activation in N-RAS knockout mice after experimental fibrosis (Fig. 4B, C). These data suggested that increased cell proliferation and matrix deposition as well as loss of cell homeostasis were characteristic of N-RAS^−/−^ mice after experimental fibrosis.

### Overactivation of JNK and AKT is a hallmark of experimental hepatotoxicity in the absence of N-RAS

The best-characterized RAS effectors are protein kinases including MAPK and PI3K [4, 14]. An anti-apoptotic function for N-RAS via downregulation of JNK was previously reported, whilst additional pro-survival function for N-RAS is exerted via the PI3K/AKT pathway [15, 16]. Therefore, we first studied JNK activation by immunoblotting after experimental liver injury (Fig. 4D, E). Phosphorylation of JNK (pJNK), an indicator of their activation, was increased in livers of N-RAS^−/−^ compared to N-RAS^+/+^ mice, after either CCl_4_ treatment or BDL surgery. Specifically, JNK1 and JNK2 overactivation was characteristic of livers from N-RAS^−/−^ mice compared to those from N-RAS^+/+^ counterparts, in both models of experimental hepatotoxicity.

As mentioned earlier, PI3K/AKT is another RAS effector, which plays an important role in RAS-mediated cell survival [14]. After chronic CCl_4_ or BDL challenge, augmented levels of phosphorylated AKT (pAKT) was detected in liver extracts of N-RAS^−/−^ compared with N-RAS^+/+^ mice, where only mild activation of AKT was observed (Fig. 4D, E). Altogether, these results indicate that hyperactivation of JNK1/2 and AKT pathways occur in the absence of N-RAS during experimental liver injury and fibrosis.

### Impact of N-RAS deficiency in acute liver injury in mice and in hepatocytic cells in vitro

**Fig. 5:**
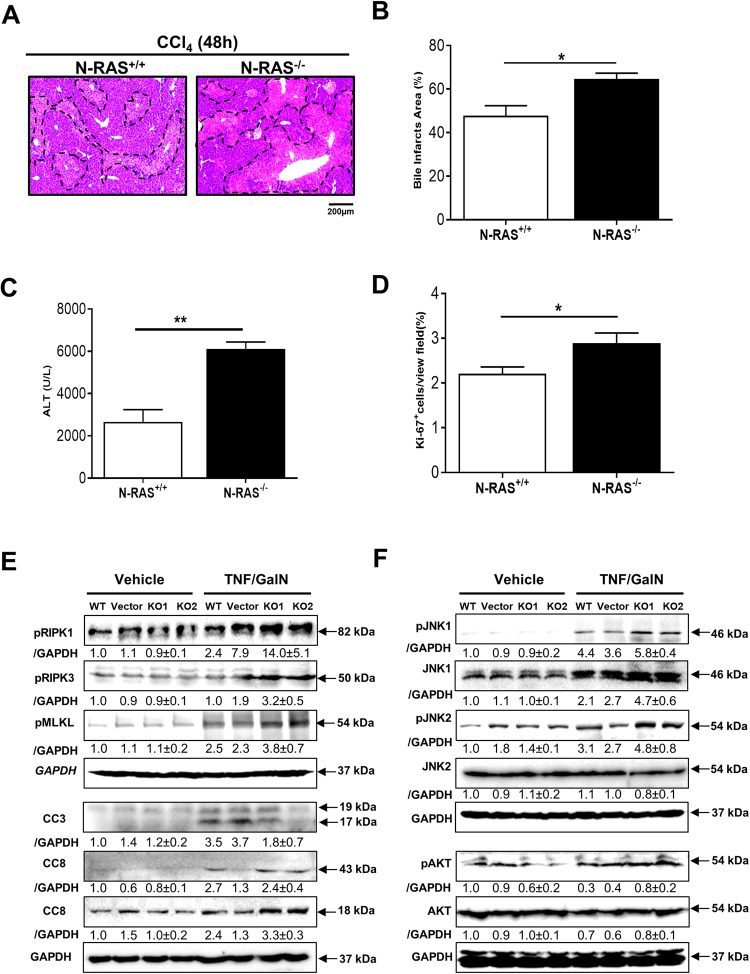
Impact of N-RAS deficiency in acute liver injury in mice and in hepatocytic cells in vitro. **A**, **B** Representative H&E staining of liver sections (**A**) and quantification of necrotic foci (**B**) area from N-RAS^+/+^ and N-RAS^−/−^ mice, after 48 h acute CCl_4_. Dotted areas denote necrotic foci. **C** Serum levels of ALT were determined in the same samples. **D** Immunofluorescence against Ki-67 was performed in N-RAS^+/+^ and N-RAS^−/−^ mice, after 48 h CCl_4_ and positive cell quantification was calculated and graphed. **E** HepG2 cells were knocked-out for N-RAS using CRISPR/Cas9 and treated with TNF/GalN or vehicle for 48 h. Protein expression was studied in cell lysates of untreated (WT), vector or knocked-out cells (KD1, KD2) by Western Blot using antibodies against pRIK1/3, pMLKL, CC3, and CC8. GAPDH was used as a housekeeping protein control. **F** Immunoblotting for pJNK1, JNK1, pJNK2, JNK2, pAKT, and AKT was performed with the same cell lysates. GAPDH was also used as loading control. Values are represent mean ± SEM from 4 mice per group (*p < 0.05).

To further understand the role of N-RAS in liver injury and cell death, we subsequently performed an in vivo model of acute liver injury (ALI), which consisted in mice receiving an acute treatment with CCl_4_ for 48 h. Compared to N-RAS^+/+^ counterparts, N-RAS^−/−^ livers exhibited increased biliary foci and necrosis, enhanced inflammation, elevation of serum markers of liver injury (ALT), and augmented compensatory proliferation of regenerating hepatocytes, in absence of differences in cell death measured by TUNEL between N-RAS^−/−^ and N-RAS^+/+^ mice, treated with CCl_4_ for 48 h (Fig. 5A–D, Suppl. Figure 9A–C). This set of data indicated that N-RAS deficiency not only aggravated chronic but also the acute liver injury induced by CCl_4_.

When N-RAS expression was analyzed in isolated liver parenchymal and non-parenchymal cells (Suppl. Figure 10A), the highest expression was observed in hepatocytes, and, to a lesser extent in Kupffer cells. Thus, to gain further insight into the role of N-RAS in hepatocyte death, we knocked-out N-RAS in HepG2 cells, a human hepatocyte-derived cell line. The efficiency of N-RAS knockout was tested by Western blot, with N-RAS^KO^ cells showing markedly reduction of N-RAS protein compared with untreated or treated with the vector alone (Suppl. Figure 10B). Because treatment of hepatocytes with TNF/GalN has been shown to induce JNK activation and necrotic cell death [17], we treated N-RAS^KO^ cells and corresponding controls with TNF/GalN for 24 h, and evaluated their effect on cell viability. In general, TNF/GalN-treated N-RAS^KO^ cells showed a tendency towards decreased cell viability compared to controls (Suppl. Figure 10C). In addition, upon TNF/GalN treatment, N-RAS^KO^ cells showed augmented phosphorylation of RIPK1/3 and MLKL, and increased CC8 but decreased CC3 (Fig. 5E), along with enhanced expression of phosphorylated JNK1/2, and mild increase in AKT (Fig. 5F), similarly to N-RAS^−/−^ mice subjected to experimental liver injury. Altogether, these experiments indicated that N-RAS ablation specifically in hepatocytes enhances the activation of RIPK1/3/MLKL and JNKs associated with induced necroptosis.

### Loss of N-RAS expression in patients with advanced chronic liver disease (ACLD)

**Fig. 6:**
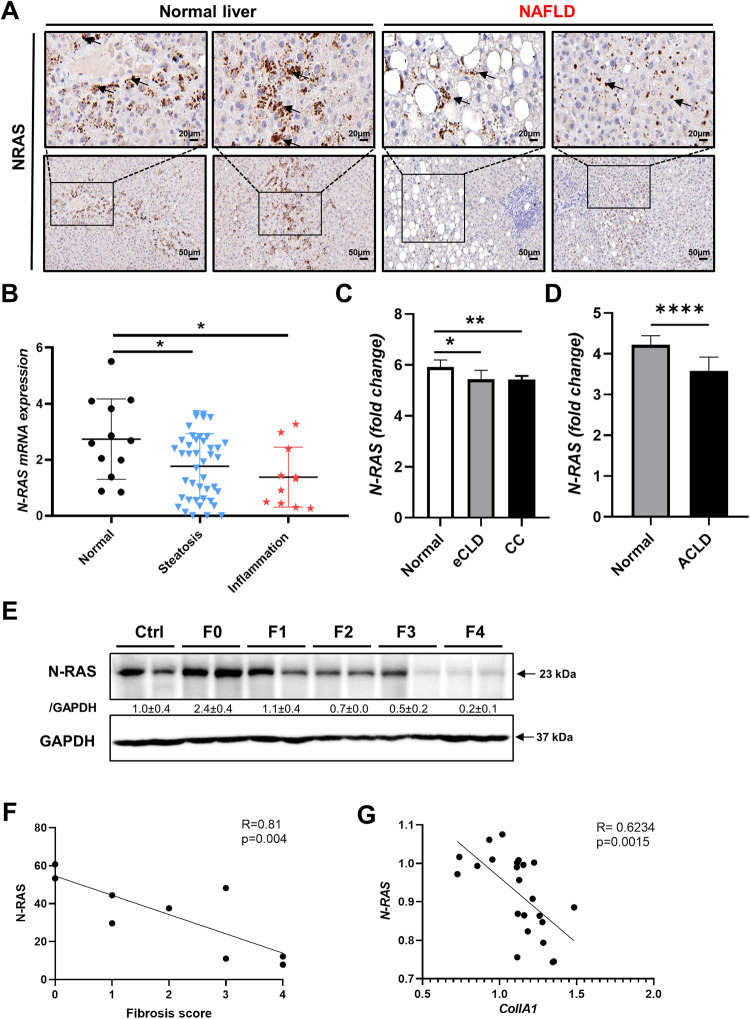
Loss of N-RAS expression in patients with advanced chronic liver disease (ACLD). **A** Immunostaining for N-RAS was tested in paraffin sections of patients with diagnosis of unaffected “normal” liver (left panel) or advanced chronic liver disease ACLD (NAFLD) with presence of steatosis and inflammation (right panel). Microphotographs were taken at 20 (top panel) and 50 µm (bottom panel), respectively. **B** mRNA expression of N-RAS was assessed in patients diagnosed with unaffected liver, steatosis, or inflammation (cohort#1). **C** N-RAS gene counts in healthy patients and in patients with early chronic liver disease (eCLD), compensated cirrhosis (CC) was calculated and normalized to the healthy group, and graphed (cohort#2). **D** N-RAS gene counts in control patients and in patients with ACLD was calculated and graphed (cohort#3). **E** Immunoblot for N-RAS was performed in fine needle liver biopsies of patients with fibrosis (Vall d’Hebron cohort). GAPDH was used as the loading control. **F** Spearmen correlation of mean N-RAS with the fibrosis score. **G** Spearmen correlation of N-RAS with collagen IA1 deposition. Values represent mean ± SEM (*p < 0.5–****p < 0.0001).

To gain insight into the relevance of N-RAS in ACLD, we first analyzed N-RAS protein expression by IHC, in affected and unaffected (healthy) areas of liver explants from patients with NAFLD/NASH (Suppl. Table 1, cohort#1). In contrast to normal liver sections where N-RAS expression was clearly distributed within the hepatocyte cytoplasm, loss of N-RAS was characteristic of patients with histologically diagnosed steatosis and inflammation, and high NAS score (Fig. 6A, Suppl. Figure 11A, B). Next, N-RAS mRNA expression was analyzed, and the results validated the IHC analyses. Significant loss of N-RAS, but not of other RAS genes such as K-RAS, was a hallmark in livers of patients with steatosis and inflammation, compared with healthy tissue (Fig. 6B, Suppl. Figure 11C). To confirm these data, we checked N-RAS counts on gene arrays of other cohorts of patients. Significantly decreased mRNA expression of N-RAS was observed in patients with early chronic liver disease (eCLD) with F2/F3 fibrosis score (Fig. 6C, cohort#2 [18]), compensated cirrhosis (CC), and with ACLD (cohort#3 [19]) (Fig. 6D).

Steatosis and inflammation are closely related to the development of fibrosis. Thus, we next evaluated protein levels of N-RAS in patients diagnosed with fibrosis (Suppl. Table 2, cohort#4). Interestingly, we observed progressive loss of N-RAS expression in late fibrotic stages F3-F4 (Fig. 6E). In fact, the levels of N-RAS negatively correlated with the fibrosis score, Collagen IA1 levels and with the FIB-4 score (Fig. 6F, G, Suppl. Figure 11D). Collectively, these data indicates that loss of N-RAS expression is a hallmark of the progression of chronic liver disease.

## Discussion

RAS proteins share a high degree of homology and are ubiquitously expressed. Despite this, increasing evidence indicates the existence of both shared and specific functions for each Ras isoform.

Previous data indicating a role for the N-RAS isoform in fibrogenesis [7], particularly in kidney fibrosis [8], prompted us to investigate the potential role of N-RAS in chronic liver disease, including fibrogenesis. For this purpose, we used a widely accepted model of ACLD, the toxic model CCl_4_ administration, which leads to hepatotoxic damage, inflammation, and fibrosis [11]. CCl_4_ administration in wild-type mice results in histopathological features of NASH with increased serum ALT and liver hydroxyproline. Therefore, it is a useful preclinical tool for modeling aspects of NASH [20] lacking metabolic alterations that typically accompany NAFLD/NASH etiology in humans. As a second hepatotoxic model we used BDL, which leads to accumulation of the bile in the liver, causing hepatic injury, inflammation and, ultimately, fibrosis [11].

Interestingly, N-RAS deficiency in both the CCl_4_ and BDL models of chronic liver disease resulted in exacerbated liver injury, as indicated by increased periportal necrotic foci, increased levels of serum markers of liver damage, accumulation of lipid peroxides, hepatocyte death and compensatory proliferation. Aggravated liver injury was accompanied by augmented fiber deposition, bridging fibrosis, collagen IA1 and ECM deposition, which suggest enhanced activation of HSCs; along with increased infiltration of immune cells in the damaged liver. Taken together, these data suggest that N-RAS signaling may act as a negative regulator of induction and progression of liver injury and fibrosis.

Interestingly, the analysis of the gene array revealed that N-RAS expression increased in wild-type mice subjected to chronic hepatotoxic damage, suggesting that N-RAS may be a signaling component of the ensuing repair response to liver damage; with this role being relevant at early stages of hepatic injury, as suggested by the exacerbated liver damage and fibrosis observed also in N-RAS^*−/−*^ mice in the acute CCl_4_-induced model of liver injury. Moreover, downregulation of genes that function to maintain hepatocyte homeostasis alongside with upregulation of genes related with immune infiltration and ECM deposition, and activation of JNK signaling was characteristic of N-RAS-deficient mice, after CCl_4_ or BDL. Collectively, these results suggest that N-RAS may protect the hepatocyte from induced injury and death, thus preventing overt inflammation and fibrosis to occur.

Apoptosis and programed necrosis/necroptosis are the major types of cell death in liver disease, with Caspase-8 representing a central switch that directs cell death towards apoptosis or necroptosis [13]. In fact, our data indicate that majorly CC3 (apoptosis) and, to a lesser extent, RIPK1/3/MLKL (necroptosis) contribute to cell death after both CCl_4_ and BDL in wild-type mice. In contrast, N-RAS-deficient mice subjected to hepatotoxic insult display less CC3 but more CC8, compared to wild-type counterparts. Moreover, the mode of cell death in response to the injury seems to be switched from apoptosis to necroptosis in N-RAS^*−/−*^ mice, which displayed a significant activation of RIPK1/3 and the pseudo-kinase MLKL. Thus, it is tempting to speculate that increased hepatocyte necroptosis associated to the N-RAS deficiency might ultimately lead to exacerbated hepatic inflammation and fibrosis, although controversial results have been obtained up-to-date with necroptosis inhibitors in preclinical models of inflammation/fibrosis [21].

Active RAS leads in turn to activation of MAPKs. MAPKs are important intracellular signal transduction systems and participate in a series of physiological and pathological processes, including cell growth, differentiation, and apoptosis [22]. One of the most prominent members of MAPK family is the c-Jun-N-terminal kinases (JNK). Our results show that N-RAS deficiency was associated with JNK overactivation upon hepatocyte injury, both in vivo (N-RAS^*−/−*^ mice) and in vitro (N-RAS-deficient HepG2 cells). In line with this, the function of N-RAS preventing cell death by attenuating JNK levels was reported [15], and JNK activation induced by UV was observed in cells where N-RAS was knocked-down [16]. Moreover, HNE, an aldehydic end product of lipid peroxidation, was reported to directly interact with JNKs and induce their activation in HSCs [23]. Of note, HNE was increased in CCl_4_-treated N-RAS^*−/−*^ mice compared to wild-type counterparts, suggesting an additional link between N-RAS and JNK activation via HNE. Collectively, these findings support the notion that N-RAS deficiency exacerbates induced liver damage partly by overactivation of the JNK signaling pathway.

A cell survival function for N-RAS through the PI3K/AKT pathway has also been described [15]. In contrast, we observed increased activation of AKT in the liver of N-RAS^*−/−*^ mice subjected to hepatic injury, but only a very mild activation in human HepG2 cells upon N-RAS ablation. Conflicting results have been obtained in N-RAS knockout cells and with inhibitors of AKT signaling. On the one hand, phosphorylation of AKT at T306, was clearly deregulated in the absence of N-RAS, while ERK activation was barely affected [15]. Moreover, selective effector-binding domain mutants of N-RAS showed that N-RAS promotes cell survival independently of AKT. In fact, AKT activation is observable in N-RAS-deficient fibroblasts [24]. Overall, these data support the notion that, while JNK overactivation in N-RAS deficiency might trigger cell death, AKT activation might be related to the increase in ECM synthesis [24, 25].

In the microarray analysis, we observed that N-RAS deficiency alters hepatocyte homeostasis in the injured liver. These results were coincident with the predominant expression of N-RAS in isolated hepatocytes *versus* other hepatic cell types. In addition, HepG2 cells with ablated N-RAS exhibited altered cell death response. It is well-known that hepatocyte death provides signals for the development of liver fibrosis, with convincing evidence showing that hepatocyte death directly activates HSCs [26].

It is well-known that gene upregulation and protein overexpression of N-RAS is a good prognostic marker of clinical HCC [27]. In contrast, a reduction in the mRNA expression and microarray counts of N-RAS was a hallmark of chronic liver disease in the databases analyzed in the present study. N-RAS in liver samples of patients with NASH undoubtedly indicated a progressive loss of this protein during the course of liver fibrosis, which was in line with the exacerbated fibrosis observed in mice deficient for N-RAS upon induced liver damage. Moreover, N-RAS immunostainings revealed strongly decreased N-RAS expression in patients with ACLD, and specifically those with NASH. To any extent, the differences in the clinicopathological characteristics of patients might explain increased N-RAS expression at very late stages, when cancer sets in.

Thus, it is tempting to speculate that modulation of N-RAS signaling in hepatocytes would impact the initiation, development, and resolution of liver injury and fibrosis; and the progression to ACLD. Yet, a role for N-RAS in liver cells other than hepatocytes [28–31] with relevance to chronic liver disease, cannot be presently excluded and deserves further investigation.

In conclusion, our study identifies a novel role for N-RAS as a negative regulator of the development of liver injury and fibrosis, partly by critically protecting the hepatocyte from necroptotic cell death. In addition, our findings regarding the loss of hepatic N-RAS expression in patients with fibrotic liver disease suggest that N-RAS may be an interesting biomarker of progressive liver damage and fibrosis, with prognostic value. Moreover, our study highlights the possibility of specifically targeting N-RAS signaling [32–34] as a novel therapeutic intervention to control the progression and ameliorate the severity of chronic liver disease.

## Materials/subjects and methods

### Experimental models of liver fibrosis

Mice were housed in the Animal Facility at the Faculty of Biology of the Complutense University (UCM). Mice were maintained in a 12:12 h light/dark cycle with free access to food and water. N-RAS^+/+^ and N-RAS^−/−^ mice were bred on a C57BL/6J genetic background as previously described [35]. N-RAS ablation was confirmed at DNA level by PCR analysis of tails from newborn mice (Suppl. Figure 1A).

Chemically-induced liver injury was performed in 8–10-week-old mice by using an i.p. administration of CCl_4_, diluted in corn oil. A single dosage of CCl_4_ (1.2 mg/kg b.w.) was given in order to induce acute liver injury (ALI). In addition, mice were chronically challenged with CCl_4_ (0.6 mg/kg b.w.), two times per week, for a period of 28 days. Corn oil was used as vehicle. All mice were sacrificed 48 h after the last injection.

Bile duct ligation (BDL) was performed to induce cholestatic liver injury as previously described [36]. Briefly, 8–10-week-old male mice were anesthetized with a ketamine combination solution (1% ketamine/0.1% xylazin combination, diluted in 0.9% NaCl) with a dosage of 0.1 mL/10 g b.w. *i.p*. Subsequently, midline laparotomy was performed to expose the common bile duct and two knots were ligated with 6–0 sutures. Sham operation was performed by laparotomy and soft touching the general bile duct of control mice. All mice were sacrificed 28 days after BDL.

### N-RAS ablation in human hepatocytes

HepG2 cells were cultured in Dulbecco’s modified Eagle’s medium (DMEM) high glucose medium, supplemented with 20% fetal calf serum, 1% penicillin/streptomycin, 2 mM L-glutamine, and 0.1 mM non-essential amino acid. We applied CRISPR/Cas9 genome engineering to knock-out N-RAS in HepG2 cells, as previously described [37]. Briefly, two different sgRNA sequences were designed based on the N-RAS gene sequence. Cells were seeded onto six-well plates with 1 × 10^5^/well. The transfection was performed when the cells reached about 70–80% confluence. 0.5 × 10^6^/well of lentivirus was added to the medium. Antibiotic selection was performed at 72 h after transfection, HepG2 cells were selected using puromycin at a concentration of 0.25 μg/mL. The selected cells were counted and seeded onto 96-well plate, and single-cell colonies were obtained after 2 weeks. The knock-out was confirmed by Western blot.

Combined TNF (30 ng/mL) and D-Galactosamine (GalN, 25 mM) treatment for 24 h was used to induce acute toxicity in Hep-G2, an immortalized cell line of human hepatocytes. HepG2 cell viability was determined by cell counting kit-8 (CCK-8) assay method according to the manufacturer’s protocol. Briefly, 1 × 10^4^ cells were plated in a 96-well plate with 100 μL medium. After TNFα/GalN treatment, 10 μL of CCK-8 solution was added to each well, followed by incubation at 37 °C for 2 h. Absorbance at 450 nm was measured to evaluate cell viability.

### Patient cohorts

This multicentric study was composed of several cohorts. Cohort#1 consisted of 91 consecutive and prospective subjects recruited for a bariatric intervention in the Virgen de la Arrixaca University Hospital (Murcia, Spain) (Supp. Table 1). Inclusion criteria were age range of 18–65 years and obesity of more than 5 years of duration with a body mass index (BMI) ≥ 40 kg/m^2^ or ≥35 kg/m^2^ with significant obesity-related comorbidities. Exclusion criteria were set based on evidence of other causes of liver disease other than NAFLD/NASH, including viral hepatitis, medication-related disorders, autoimmune disease, hemochromatosis, Wilson’s disease, familial/genetic causes, or a previous history of excessive alcohol use (>30 g daily for men and >20 g daily for women) or treatment with any drugs potentially causing steatosis, such as tamoxifen, amiodarone, and valproic acid. Hepatic tissue from histologically non-affected livers (no steatosis, inflammation and/or fibrosis) was used as controls (labeled as “Normal liver” in the figures).

A recently characterized cohort (cohort#2) of 13 patients [18] encompassing the whole spectrum of chronic liver disease (CLD) stages due to any liver etiology ranging from patients with F1/F2/F3 fibrosis, named early-CLD to compensated cirrhosis (CC) was also used.

Cohort#3 consisted of 13 patients with advanced-CLD that underwent liver transplantation and control patients with no suspected CLD, who underwent liver resection due to colorectal carcinoma (CRC) metastasis [19].

Cohort#4 included 93 patients with biopsy-proven NAFLD/NASH samples at different stages of steatohepatitis and fibrosis progression. Percutaneous liver biopsy was indicated and carried out according usual clinical practice for NAFLD/NASH diagnose confirmation and disease staging (Suppl. Table 2). Liver samples from histologically normal livers without signs of steatosis or fibrosis were selected as healthy controls.

### Statistics

All data are shown as mean and error bars are shown as standard error of the mean (SEM). Differences between experimental groups were assessed by two-way ANOVA, followed by Tukey’s multiple comparisons test, t-test Independent samples 2 tails, and Pearson Chi-Square 2 sided. GraphPad Prism software (version 6.01) was employed to perform all statistical analyses.

## Supplementary information

Suppl. Figures and Tables

Suppl. File uncropped WBs

Checklist

## Data Availability

The experimental data sets generated and/or analyzed during the current study are available from the corresponding author upon reasonable request. No applicable resources were generated during the current study. Affimetrix microarray data have been deposited with the NCBI Gene Expression Omnibus under accession number GSE236915.
