# Supplementary material for: Neuroblastoma RAS viral oncogene homolog (N-RAS) deficiency aggravates liver injury and fibrosis
Source: Cell Death Dis. 2023 Aug 10;14(8):514. doi: 10.1038/s41419-023-06029-y (PMC10415403; doi:10.1038/s41419-023-06029-y)
Supplement: Supplementary file 1 — Suppl. Figures and Tables [file 41419_2023_6029_MOESM1_ESM.pdf]

SUPPLEMENTARY MATERIAL to:

Neuroblastoma RAS viral oncogene homolog (N-RAS) deficiency aggravates liver injury and fibrosis

Kang Zheng, Fengjie Hao, Sandra Medrano-Garcia, Chaobo Chen, Laura Morán-Blanco, Sandra Rodríguez-Perales, Raúl Torres-Ruiz, María Isabel Peligros, Javier Vaquero, Rafael Bañares, Manuel Gómez del Moral, José R. Regueiro, Eduardo Martínez-Naves, Rocío Gallego-Durán, Douglas Maya, Javier Ampuero, Manuel Romero-Gómez, Albert Gilbert-Ramos, Sergi Guixé-Muntet, Anabel Fernández-Iglesias, Jordi Gracia-Sancho, Mar Coll, Isabel Graupera, Pere Ginès, Andreea Ciudin, Jesús Rivera-Esteban, Juan M. Pericàs, María Dolores Frutos, Bruno Ramos Molina, José María Herranz, Matías A. Ávila, Yulia A. Nevzorova, Edgar Fernández-Malavé, Francisco Javier Cubero

SUPPLEMENTARY METHODS

Isolation of hepatic cell types

For the isolation of different hepatic cell types (hepatocytes, HSCs, KCs/LECs) the solutions in **Suppl. Tables 3&4** are required. For the density gradient medium, the following solutions were prepared before starting the perfusion of the liver: Nycodenz® 1:5.18 g Nycodenz in 15 ml GBSS-A and Nycodenz® 2: 3.63 g Nycodenz in 25 ml GBSS-A. First, all required solutions were pre-warmed in a water bath at 37°C and a winged infusion set was

mounted onto the silicone tube of a peristaltic pump. The peristaltic pump was calibrated with DPBS to obtain a laminar flow rate of approximately of 5.0ml/min to perfuse the liver. Next, the silicone tube was equilibrated with solution A and all air bubbles released. In the next step, the mouse was sacrificed by an overdose of isoflurane anesthesia and the abdomen was carefully opened along the linea alba. The intestines were moved out of the abdominal cavity to the right side of the animal in order to expose the vena cava inferior. Carefully, the catheter (20G or 26G) was inserted into the vena cava inferior and after proof of proper catheter placement the perfusion was started. After initiating the perfusion, the portal vein was immediately opened to allow a constant flow rate through the liver and subsequently a small piece of the liver was cut off for further analysis. A successful flushing of the liver is indicated by a loss of color of the liver. The liver was perfused with each solution (A,B and C), separately for 5 min or 25 ml. After the final solution C, the perfusion was stopped and the liver was dissected and transferred into solution D and incubated for 20 min at 37°C. To assure a proper digestion of the liver, solution D was inverted several times during the incubation time. Next, solution D containing the digested liver was filtered through a 70 µm cell strainer and the filtered solution was centrifuged at 50x g for 1 min at 4°C. The pellet containing hepatocytes was kept for further analysis and the supernatant was transferred to a new falcon and subsequently centrifuged at 720x g for 8 min at 4°C. The supernatant was removed and the pellet was

resuspended in 10 ml GBSS-B solution containing 150 µl DNAse I stock solution and the falcon was filled up to 50 ml with GBSS-B solution. In the next step, the solution was centrifuged at 720x g for 8 min at 4°C and the supernatant was removed. Next, the pellet was resuspended in 10 ml GBSS-B containing 150 µl DNAse I stock solution. In addition, 24 ml GBSS-B and 14 ml of the Nycodenz® 1 solution was added and the solution was carefully mixed. Further, 4 ml of the Nycodenz® 2 solution was transferred into six 15 ml falcon tubes. Next, 8.3 ml of the mixed cell solution containing GBSS-B, DNAse and Nycodenz® 1 was carefully laid onto the Nycodenz® 2 solution and the GBSS-B solution was used to gently overlay the cell suspension to obtain a final volume of 15 ml. Consequently, the gradient solution was centrifuged without brakes at 3000x g for 20 min at 4°C. After the centrifugation step, HSCs are found in the upper gradient phase as a white ring and KCs/LECs are found in the lower gradient phase. Each cell type was carefully transferred to a new falcon and washed with GBSS-B solution and subsequently centrifuged at 720x g for 8 min at 4°C to pellet the cells. The supernatant was removed and subsequently frozen in liquid nitrogen and stored at -80°C for further RNA isolation.

Liver leucocyte isolation and flow cytometry

Livers were dissected, cut into small segments, and digested with 0.5mg/ml of Liberase TM (Roche) in RPMI 1640 (Gibco) for 40 minutes, with agitation at

37^oC. The digested tissue was put on ice and 100µl of 0.1M EDTA was added to stop digestion, followed by passing through a 40-µm mesh cell strainer (Corning) and centrifugation for 6 min at 170g to remove hepatocytes. Supernatant was collected, centrifuge at 300g for 10 minutes, and the pellets treated with ACK lysis buffer (Thermo Fisher Scientific) to remove red blood cells. After washing, cells were resuspended in RPMI 1640, passed again through a cell strainer, centrifuged, and resuspended in RPMI. Cell counting was performed using a Neubauer chamber after staining with trypan blue to exclude dead cells. For flow cytometry analysis, cells were resuspended in FACS buffer (PBS containing 5% bovine serum albumin and 0.1% sodium azide), followed by Fc receptor blocking with anti-CD16/CD32 antibodies (BD Biosciences). Cells were then stained with fluorochrome-conjugated antibodies to CD45, CD3, TCRβ, TCRδ, CD4, CD8, NK1.1 and CD19; all from BD Biosciences. Data were acquired with a BD FACSCalibur flow cytometer and analyzed using FlowJo™ v10 Software (BD Biosciences).

Quantitative real-time PCR (qPCR)

Trizol reagent (Invitrogen, Karlsruhe, Germany) was used to isolate total RNA from liver tissues. Reverse-transcription was performed using an Omniscript RT Kit (Qiagen Iberia SL, Barcelona, Spain)) according to the instructions. Relative quantitative gene expression was measured via real-time PCR using a 7900HT Fast Real Time PCR System with SDS software 2.3 (Applied

Biosystems, Foster City, CA) and a SYBR Green PCR Kit (Invitrogen). GAPDH expression was used as internal standard. Primers are available upon request.

Histological evaluation and immunofluorescence staining

Hepatic tissue was fixed in 4% paraformaldehyde (PFA) immediately after extraction, embedded in paraffin, sectioned and stained for H&E or Sirius red. Samples were reviewed by a pathologist blinded to and who analyzed the degree of liver injury. The percentage of Sirius red (SR) area fraction in all animals was quantified on 10 low-power (magnification, X10) fields per slide, using NIH ImageJ® software (http://rsbweb.nih.gov/). Immunohistochemistry for 4-HNE (Abcam, Cambridge, UK) on paraffin sections was performed. Briefly, liver sections were deparaffinized with xylene and rehydrated with serially descending percentages of ethanol. The sections were then boiled in 10 mM sodium citrate acid buffer (pH: 6,0) in order to retrieve the antigen followed by incubation with 0.3% Tween20. Afterwards, sections were subjected to BLOXALL blocking solution (Palex Medical SA, Sant Cugat del Vallés, Spain). Non-specific sites were blocked with normal horse serum solution. Then, the sections were incubated overnight with primary antibodies diluted in PBT (PBS, 0.1% BSA, 0.05% TritonX-100) at 4°C in a humid box. The next day, slides were incubated with secondary for 1h at RT in a humidifying box. The signal was developed with Diaminobenzidine (DAB,

peroxidase substrate kit) (Palex Medical SA). Afterwards, the sections were counterstained using hematoxylin and mounted with Roti-Histokit. For the immunofluorescence staining, frozen cryosections were incubated with Ki-67 (Santa Cruz, Heidelberg, Germany), Collagen IA1 (Werfen, Barcelona, Spain), CD45 (BD Biosciences, San Agustín de Guadalix, Madrid, Spain), CD11b (BD Biosciences), F4/80 (Abcam) or *in situ* cell death detection kit (Roche, Mannheim, Germany) and incubated with fluorescence labeled secondary antibodies (AlexaFluor 488, Invitrogen). All fluorescence-labeled cryosections were analyzed and documented using an Imager Z1 fluorescence microscope together with Axiovision software (Carl Zeiss, Jena, Germany).

Immunoblot analysis

Liver tissues were homogenized in ice cold NP40-Buffer containing 50 mM Tri- HCl (pH 7.5), 150 mM NaCl, 0.5% NP-40 and 50 mM NaF freshly supplemented with Complete Mini (Merck, Madrid, Spain), PhosphoSTOP (Merck), 1 mM orthovanadate and 1 mM Pefablock. Protein concentrations were determined by BIO-RAD protein assay (Biorad, Madrid, Spain). Samples were separated by SDS-PAGE and transferred to a cellulose membrane and probed with antibodies for PCNA (Dianova GmbH, Hamburg, Germany), α-SMA (Invitrogen, Carlsbad, CA), Ki-67 (Abcam), Phospho-MLKL (Abcam), Phospho-RIPK1 (Abcam), CC3 (Werfen), CC8 (Werfen), Phospho-RIPK3

(Abcam), Phospho-JNK (Werfen), Phospho-JNK1 (Werfen), Phospho-JNK2 (Werfen), Phospho-AKT (Werfen), JNK (Werfen), JNK1 (Abcam), JNK2 (Werfen), AKT (Werfen) and GAPDH (Biorad). Anti-rabbit-HRP (Biorad) and anti-mouse-HRP (Biorad) were used as secondary antibodies.

Microarray analysis

RNA was isolated from livers using TRIzol reagent according to the manufacturer's instructions. Samples were analyzed by the Department of Genomics, Faculty of Biology, Complutense University, Madrid, Spain. The analysis is done with the Transcriptome Analysis Console (TAC) software. First, we selected the top up- and downregulated genes in the dataset based on interquartile range and then selected genes associated with parameters related to hepatic fibrosis, liver metabolism, hepatic infiltration and inflammation. These genes were then hierarchically clustered and statistically analyzed by Pearson's correlation (n = 3 per group).

Other Methodology

Serum transaminases in blood were analyzed in the Central Laboratory Facility at the Gregorio Marañón Research Health Institute at Madrid (IISGM) using automated analyzers.

SUPPLEMENTARY TABLES

Suppl. Table 1. Clinicopathological characteristic of patients in cohort#1.

NAFLD			
	**Healthy** (n=21)	**Steatosis** (n=53)	**Inflammation** (n=17)
**Age**	44.0±11.0	47.5±11.6	46.0±10.7
**BMI**	42.5±4.5	43.0±5.8	42.9±6.4
**Gender (M/F)**	20/1	36/17	14/3
**AST**	17.4±4.9	20.0±7.6[###]	21.9±9.2****
**ALT**	16.3±6.5	22.7±11.0[###]	24.5±10.9****
**Cholesterol**	157.8±26.6	168.4±34.4	177.8±33.9
**TAG**	135.2+42.6	222.4+178.8*	204.4±60.8
**HDL**	48.7±12.8	41.2±10.5*	39.5±8.3
**LDL**	85.3±22.5	89.4±32.1	97.6±34.3
**GGT**	22.7±26.9	23.8±17.0	25.9±15.7
**FIB4**	0.9±0.5	0.8±0.3	0.8±0.4
**% Fat**	46.6±3.5	43.0±6.1*	43.6±5.7
**Glucose**	84.8±21.3	108.2±34.7*	106.9±37.9**

*= Differences respect healthy group
#=Differences between Steatosis and Inflammation groups

Suppl. Table 2. Clinicopathological characteristic of patients in cohort#4 with different stages of liver fibrosis.

NASH					
	**F0** (n=4)	**F1** (n=4)	**F2** (n=3)	**F3** (n=41)	**F4** (n=41)
**Age**	54.5±7.9	65.6±9.9	57.9±21.0	68±5.9	64±9.0
**Gender (M/F)**	0/4	3/1	0/3	22/19	17/24
**AST**	35.5±6.8	69±36.8	91.7±115.5	48±34.4	51±22.3
**ALT**	28.8±8.3	54.3±29.8	57±47.0	56±49.5	57±29.7
**FIB4**	0.9±0.3	1.7±0.5	1.4±0.4	1.6±0.2	2.8±1.2
**GGT**	134.8±111.8	58.8±35.9	126.0±120.8	93.8+110.3	243.6+343.6
**Cholesterol**	218.0±45.4	211.3±28.9	225.7±22.1	181.3±33.1	201.9±46.7
**HDL**	56.5±12.2	46.5±7.0	48.3±7.4	45.1±9.0	48.7±12.6
**LDL**	135.8±37.2	124.8±16.8	143.0±19.9	102.3±25.5	112.0±35.1
**Insulin**	8.8±1.7	17.5±10.4	20.1±2.6	44.0±38.7	85.3±246.0
**Glucose**	90.8±17.6	113.5±10.8	159.0±110.3	134.0±36.0	141.0±65.9
**TGC**	139.0±35.2	174.8±31.9	152.3±62.0	173.1±85.7	164.6±110.1

Suppl. Table 3. Stock solutions for hepatic cell isolation

(mg)	**SC1**	**SC2**	**GBSS-A**	**GBSS-B**
**EGTA**	95	-	-	-
**Glucose**	450	-	495.5	495.5
**HEPES**	1190	1190	-	-
**KCl**	200	200	185	185
**Na_2HPO_4*$2H_2O$**	75.5	75.5	37.5	37.5
**NaCl**	4000	4000	-	4000
**NaH_2PO_4*H_2O**	39	39	-	-
**$NaHCO_3$**	175	175	113.5	113,5
**Phenol Red**	3	3	3	3
**$CaCl_2$*$2H_2O$**	-	280	112.5	112.5
**KH_2PO_4**	-	-	15	15
**$MgCl_2$*$6H_2O$**	-	-	105	105
**$MgSO_4$*$7H_2O$**	-	-	35	35
**H_2O to (ml)**	500	500	500	500

Suppl. Table 4. Solutions for hepatic perfusion

	**A**	**B**	**C**	**D**
**SC-1 (ml)**	50	-	-	-
**SC-2 (ml)**	-	50	50	50
**DNAse I (stock solution)**	-	-	-	500µl*
**Collagenase D (mg)**	-	-	40	40
**Pronase E (mg)**	-	16	-	26

*DNase I stock solution: 2mg/ml in GBSS-B

SUPPLEMENTARY FIGURE LEGENDS

Suppl. Fig. 1. (**A**) PCR blots of tail DNA from N-RAS$^{+/+}$ and N-RAS$^{-/-}$ mice confirmed the genotype of interest. (**B-D**) mRNA expression analysis of N-RAS (**B**), K-RAS (**C**) and H-RAS (**D**) in liver samples taken from N-RAS$^{+/+}$ and N-RAS$^{-/-}$ livers, after 28 days of CCl_4 treatment. Values are mean ± SEM (n=6-8; intragroup ****p<0.0001; intergroup $^{\#}$p<0.05-$^{\#\#\#\#}$p<0.001).

Suppl. Fig. 2. mRNA expression analysis of Collagen IA1 (**A**) and αSMA (**B**) in the same liver samples. Values are mean ± SEM (n=6-8; intragroup ****p<0.0001; intergroup $^{\#}$p<0.05-$^{\#\#\#\#}$p<0.001).

Suppl. Fig. 3. (**A**) Representative immunofluorescence staining for CD45 in N-RAS$^{+/+}$ and N-RAS$^{-/-}$ mice, after 28 days of CCl_4 treatment. (**B**) Quantification of positive cells in the same samples. (**C**) Representative immunofluorescence staining for CD11b in N-RAS$^{+/+}$ and N-RAS$^{-/-}$ mice, after 28 days of CCl_4 treatment. (**D-E**) Quantification of CD11b- (**E**) and F4/80- (**C**) positive cells in the same samples. Arrows (→) indicate positive cells. Values are mean ± SEM (n=6-8; intragroup ***p<0.001-****-p<0.0001; intergroup $^{\#\#}$p<0.01-$^{\#\#\#\#}$p<0.001).

Suppl. Fig. 4. Impact of the N-RAS deficiency on the immune cell

landscape of the liver after CCl_4-induced hepatic damage. (A-F) Leucocytes isolated from the liver of N-RAS$^{+/+}$ or N-RAS$^{-/-}$ mice, treated with CCl_4 or vehicle (corn oil), were stained with specific antibodies (**A**, TCRβ^{+} CD3^{+}; **B**, TCRβ^{+} CD3^{+} CD8^{+}; **C**, TCRβ^{+} CD3^{+} CD4^{+}; **D**, TCRβ^{+} NK1.1^{+} ; **E**, TCRβ^{+} CD3^{+}; and F, CD19^{+}) and analyzed by flow cytometry. Frequencies (%) of the indicated cell subsets are shown as mean ± SEM (n=5-8 per group). *P≤0.05, **P≤0.01, ***P≤0.001, ****P≤0.0001 (one-way ANOVA).

Suppl. Fig. 5. Macroscopic appearance of livers N-RAS$^{+/+}$ and N-RAS$^{-/-}$ livers, 28 days after CCl_4 treatment. (**B**) Liver weight (LW) versus body weight (BW) ratio from N-RAS$^{+/+}$ and N-RAS$^{-/-}$ mice, 28 days after CCl_4 was calculated and graphed. (**C**) Quantification of size of the necrotic foci in the same liver samples using H&E. (**D**) Representative immunostaining for 4-HNE of N-RAS$^{+/+}$ and N-RAS$^{-/-}$ livers, 28 days after CCl_4 treatment. Corn oil treated mice were used as controls. (**E**) Accumulation of 4-HNE was calculated and graphed. Values are mean ± SEM from 6-8 mice per group (intragroup, *p<0.05-****p<0.0001; intergroup $^{##}$p>0.01).

Suppl. Fig. 6. (**A**) Quantification of Sirius red area in paraffin sections from N-RAS$^{+/+}$ and N-RAS$^{-/-}$ livers, 28 days after BDL. (**B-E**) mRNA expression of Collagen I1A (**B**), Collagen III (**C**), α-SMA (**D**), TIMP1 (**E**) and IL-1ß (**F**) was evaluated in liver samples from N-RAS$^{+/+}$ and N-RAS$^{-/-}$ livers sacrificed 28

days after BDL. Values are mean ± SEM from 5-6 mice per group (intragroup; ***p< 0.001- ****p<0.0001; intergroup; $^{\#}$p< 0.05- $^{\#\#\#\#}$p<0.0001).

Suppl. Fig. 7. Representative immunostaining and quantification of positive cells for CD45 (**A-B**), CD11b (**C-D**) and F4/80 (**E-F**) in liver cryosections of N-RAS$^{+/+}$ and N-RAS$^{-/-}$ livers, 28 days after BDL. Values are mean ± SEM from 5-6 mice per group (intragroup; **p<0.01-***p<0.001; intergroup; $^{\#\#}$p<0.01).

Suppl. Fig. 8. (A) Macroscopic appearance of livers N-RAS$^{+/+}$ and N-RAS$^{-/-}$ livers, 28 days after BDL treatment. (**B**) Quantification of the size of necrotic foci was calculated and graphed. (**C**) Representative immunostaining for 4-HNE in N-RAS$^{+/+}$ and N-RAS$^{-/-}$ livers, 28 days after BDL. Sham-operated mice were used as controls. (**D**) Accumulation of 4-HNE was calculated and graphed. Values are mean ± SEM from 5-6 mice per group (intragroup, ***p<0.001-****p<0.0001; intergroup $^{\#}$p<0.05-$^{\#\#}$p>0.01).

Suppl. Fig. 9. (A) Representative Ki-67 immunostaining in liver cryosections of N-RAS$^{+/+}$ and N-RAS$^{-/-}$ mice, 48 h after CCl_4. **(B)** TUNEL staining was performed in the same sections. **(C)** Quantification of TUNEL-positive cells was calculated and graphed.

Suppl. Fig. 10. (A) N-RAS mRNA expression was evaluated in isolated liver

parenchymal and non-parenchymal cells of wildtype mice, (HSC: hepatic stellate cells; KC: Kupffer cells). **(B)** Knock-down of N-RAS using CRISPR/Cas9 technology was confirmed by Western blot. (**C**) Cell viability after each condition. Values are mean ± SEM (*p<0.05).

Suppl. Fig. 11. (**A**) N-RAS-positive cells per view field were quantified and represented. Values represent mean ± SEM (*p<0.5). (**B**) Representative N-RAS staining in an unaffected ¨normal¨ liver section (Healthy) and a section of a patient diagnosed with NAFLD (cohort#1). Magnification of 200µm. (**C**) mRNA expression of K-RAS was analyzed in patients diagnosed with unaffected liver, steatosis or inflammation (cohort#1). (**D**) Negative correlation of N-RAS with the fibrosis-4 (FIB-4) score is shown (Spearmen correlation; cohort#4).

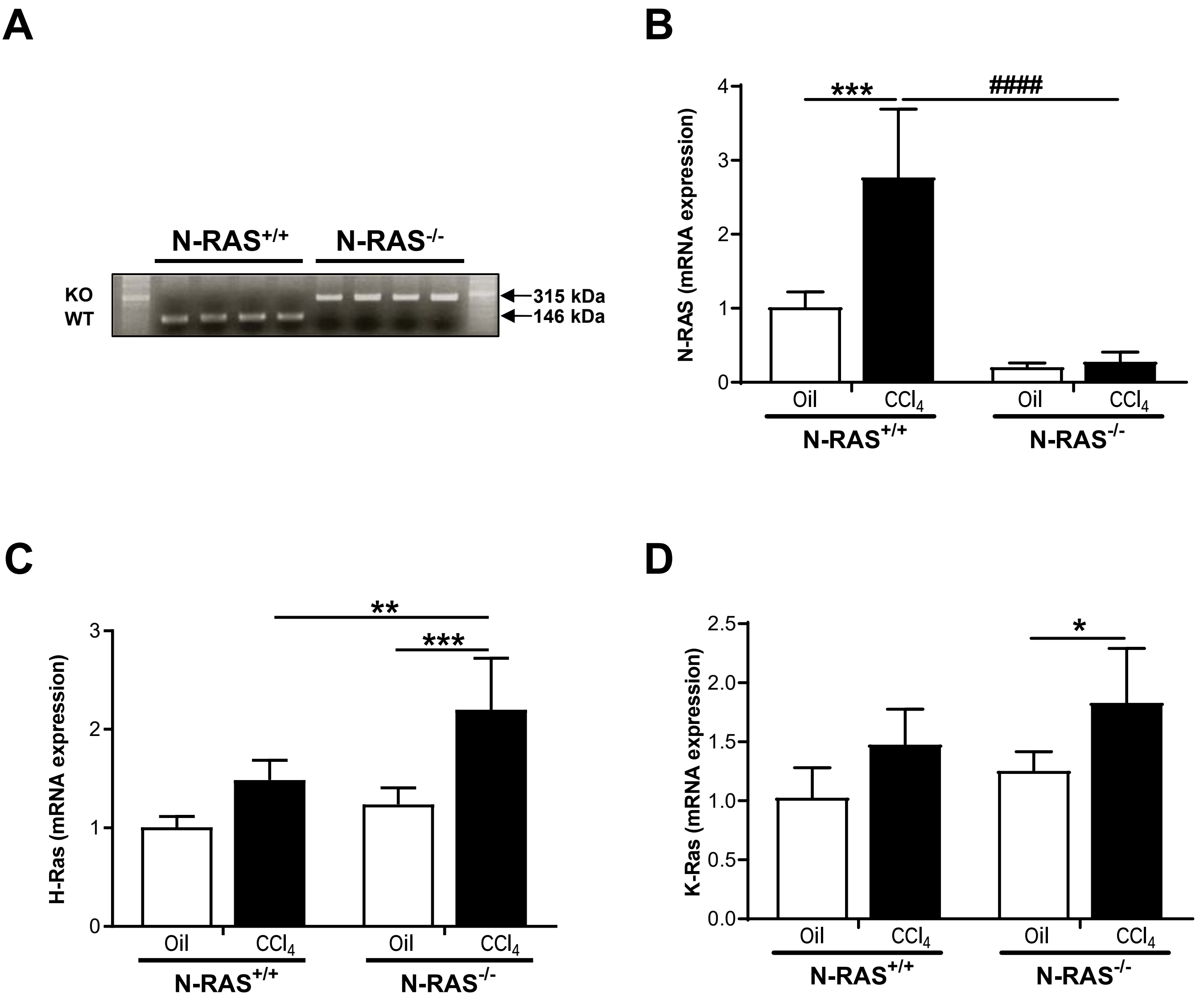
A
N-RAS+/+
N-RAS-/-
KO
WT
315 kDa
146 kDa
B
N-RAS (mRNA expression)
***
####
Oil
CCl4
N-RAS+/+
N-RAS-/-
C
H-Ras (mRNA expression)
**
***
D
K-Ras (mRNA expression)
*

Suppl. Fig. 1

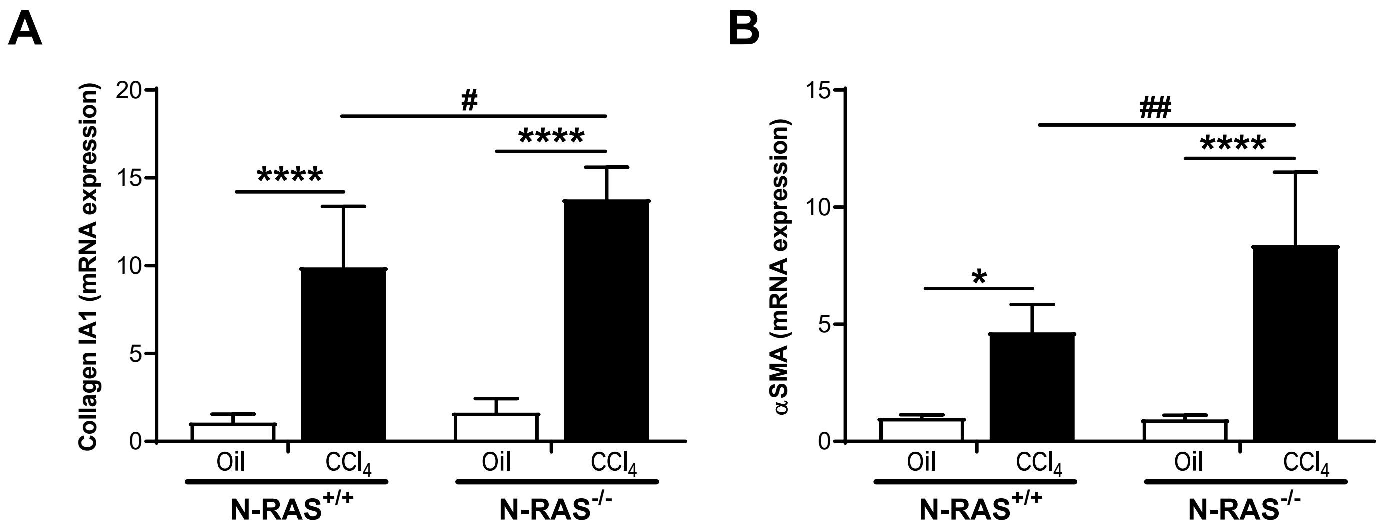
A
Collagen IA1 (mRNA expression)
20
15
10
5
0
#
****
****
Oil
CCl4
Oil
CCl4
N-RAS+/+
N-RAS-/-
B
αSMA (mRNA expression)
15
10
5
0
##
*
****
Oil
CCl4
Oil
CCl4
N-RAS+/+
N-RAS-/-

Suppl. Fig. 2

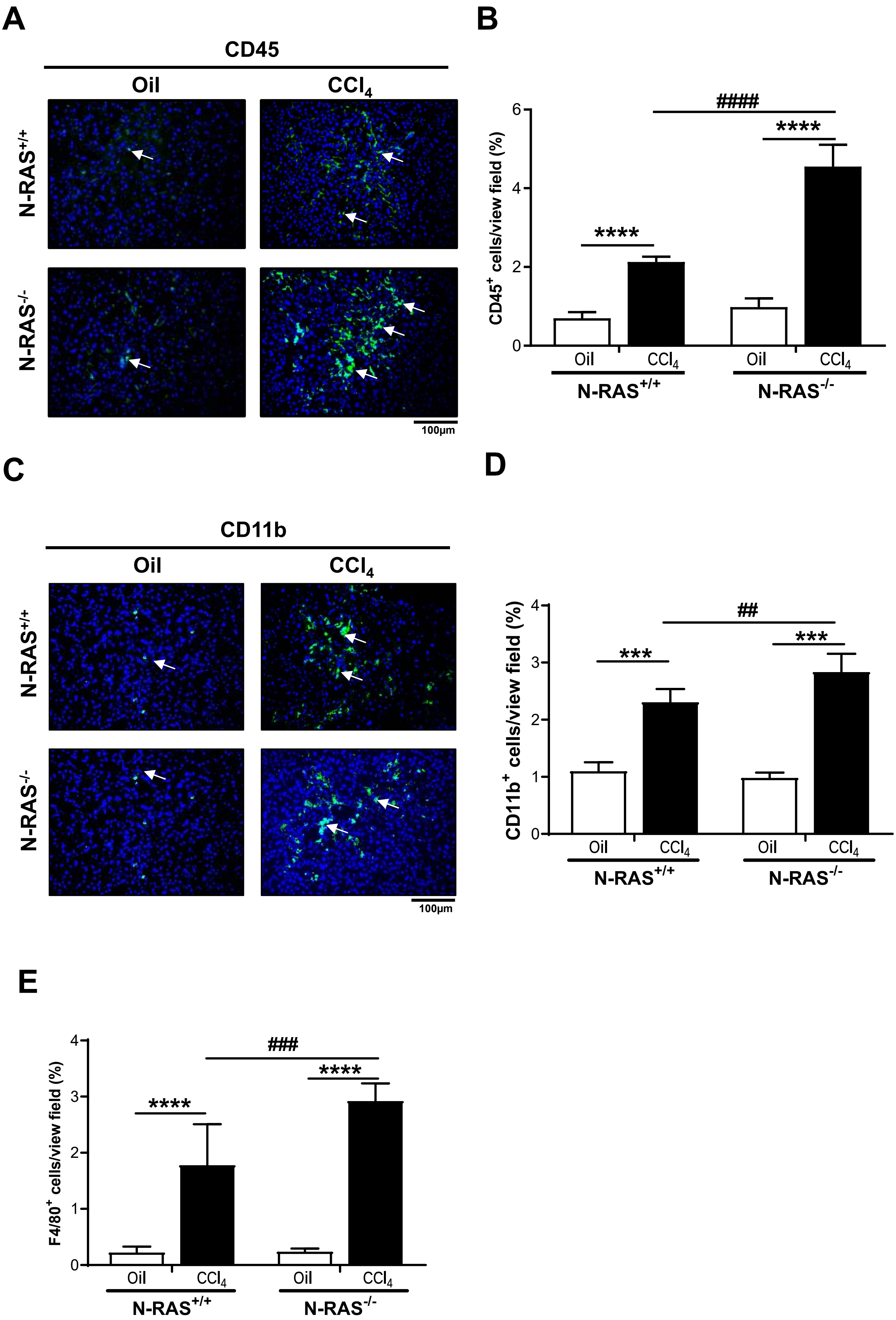
A
CD45
Oil
CCl4
N-RAS+/+
N-RAS-/-
100μm
B
CD45+ cells/view field (%)
6
4
2
0
####
****
****
Oil
CCl4
Oil
CCl4
N-RAS+/+
N-RAS-/-
C
CD11b
Oil
CCl4
N-RAS+/+
N-RAS-/-
100μm
D
CD11b+ cells/view field (%)
4
3
2
1
0
##
***
***
Oil
CCl4
Oil
CCl4
N-RAS+/+
N-RAS-/-
E
F4/80+ cells/view field (%)
4
3
2
1
0
###
****
****
Oil
CCl4
Oil
CCl4
N-RAS+/+
N-RAS-/-

Suppl. Fig. 3

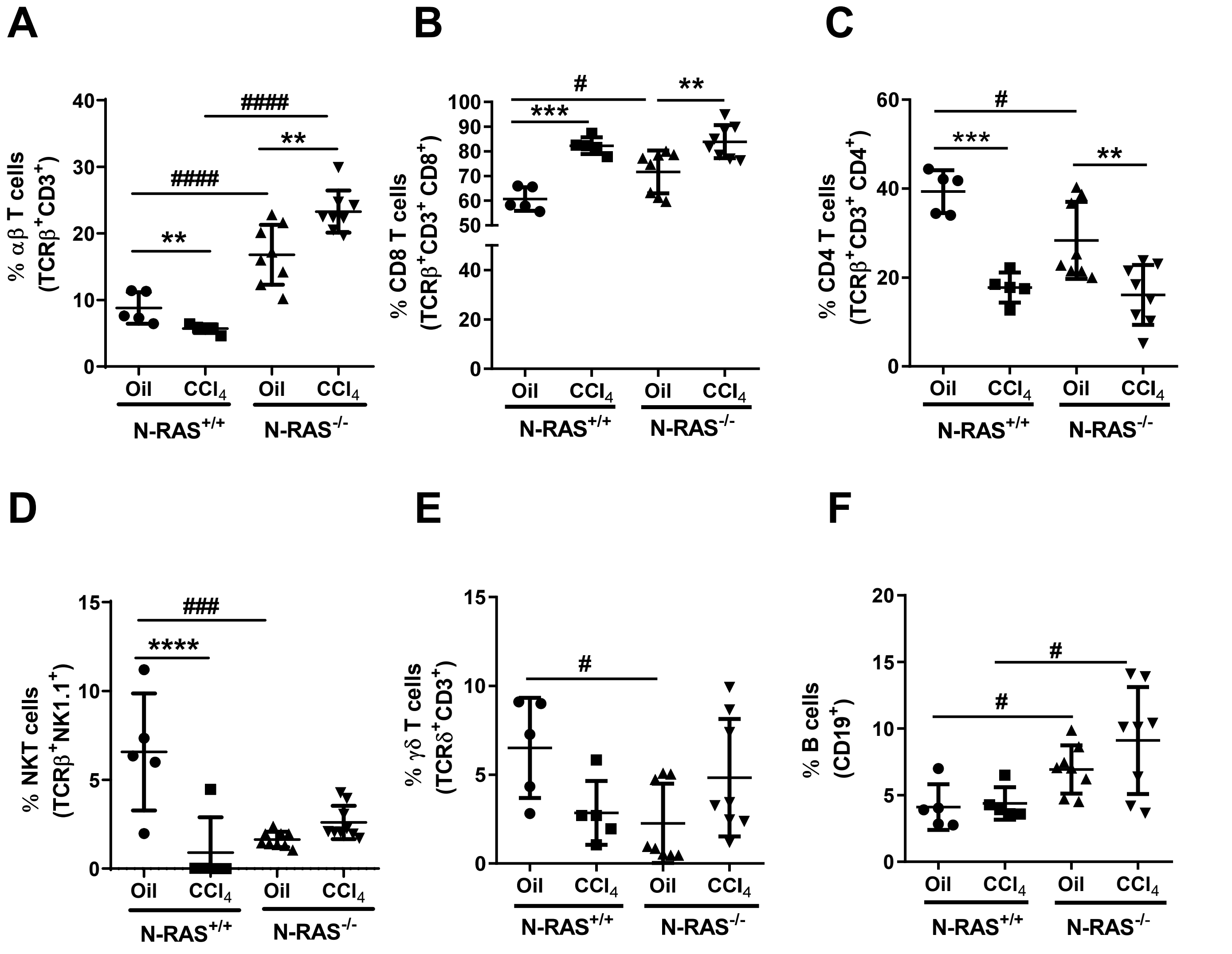
A
% αβ T cells (TCRβ+CD3+)
40
30
20
10
0
####
####
**
**
Oil
CCl4
Oil
CCl4
N-RAS+/+
N-RAS-/-
B
% CD8 T cells (TCRβ+CD3+ CD8+)
100
90
80
70
60
50
40
20
0
#
***
**
Oil
CCl4
Oil
CCl4
N-RAS+/+
N-RAS-/-
C
% CD4 T cells (TCRβ+CD3+ CD4+)
60
40
20
0
#
***
**
Oil
CCl4
Oil
CCl4
N-RAS+/+
N-RAS-/-
D
% NKT cells (TCRβ+NK1.1+)
15
10
5
0
###
****
Oil
CCl4
Oil
CCl4
N-RAS+/+
N-RAS-/-
E
% γδ T cells (TCRδ+CD3+)
15
10
5
0
#
Oil
CCl4
Oil
CCl4
N-RAS+/+
N-RAS-/-
F
% B cells (CD19+)
20
15
10
5
0
#
#
Oil
CCl4
Oil
CCl4
N-RAS+/+
N-RAS-/-

Suppl. Fig. 4

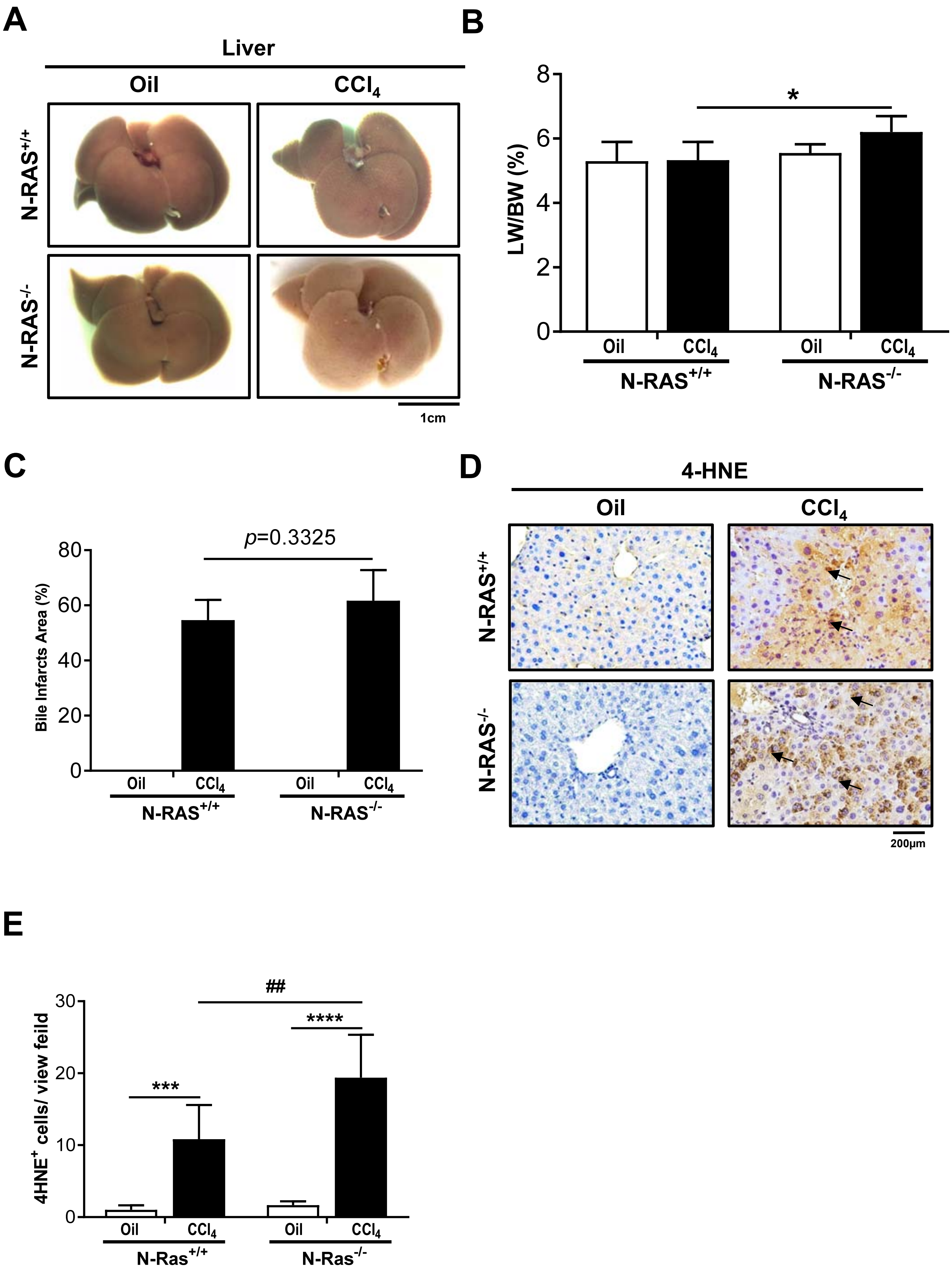
A
Liver
Oil
CCl4
N-RAS+/+
N-RAS-/-
1cm
B
LW/BW (%)
8
6
4
2
0
*
Oil
CCl4
Oil
CCl4
N-RAS+/+
N-RAS-/-
C
Bile Infarcts Area (%)
80
60
40
20
0
p=0.3325
Oil
CCl4
Oil
CCl4
N-RAS+/+
N-RAS-/-
D
4-HNE
Oil
CCl4
N-RAS+/+
N-RAS-/-
200μm
E
4HNE+ cells/ view feild
30
20
10
0
##
****
***
Oil
CCl4
Oil
CCl4
N-Ras+/+
N-Ras-/-

Suppl. Fig. 5

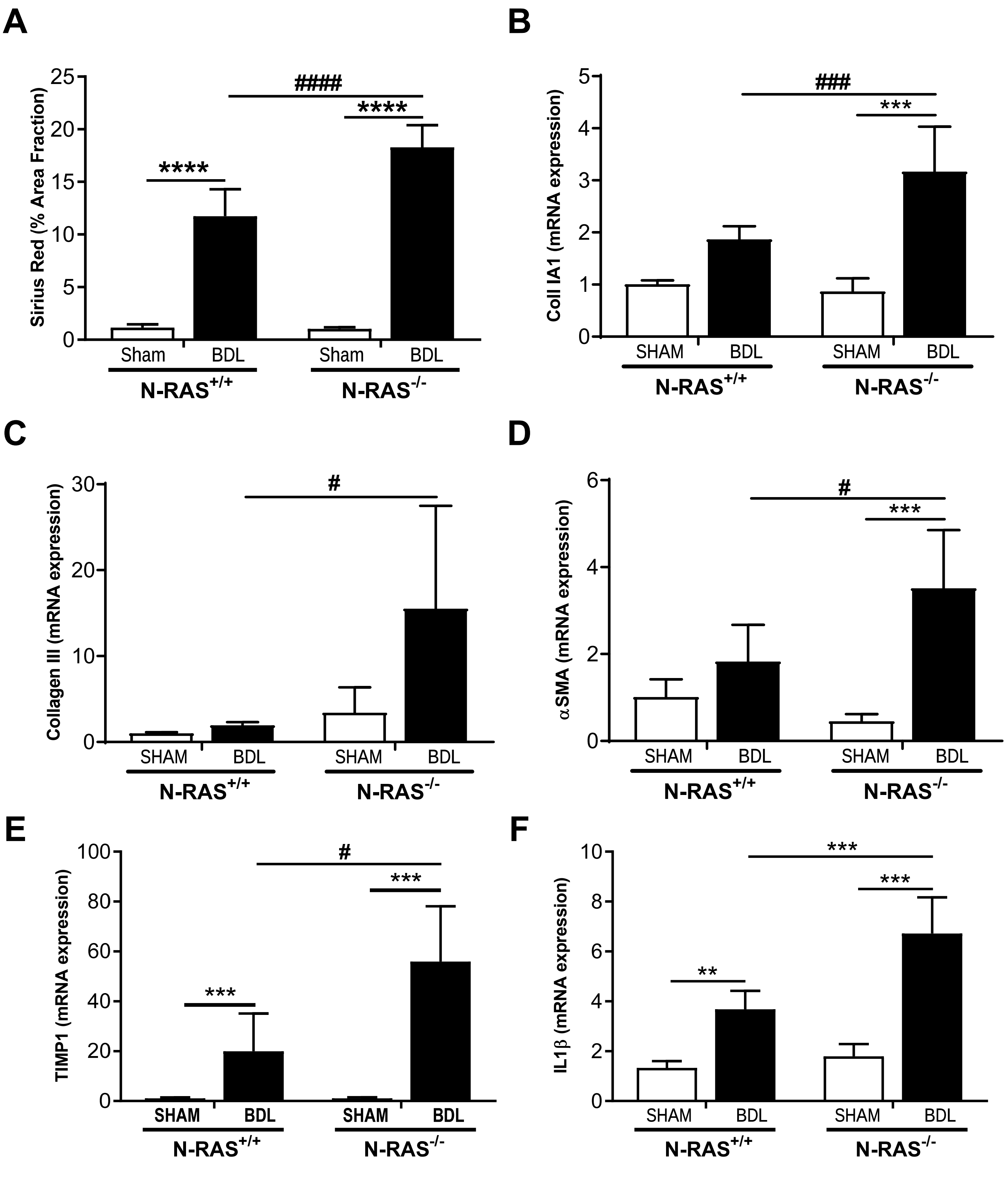
A
Sirius Red (% Area Fraction)
25
20
15
10
5
0
####
****
****
Sham
BDL
Sham
BDL
N-RAS+/+
N-RAS-/-
B
Coll IA1 (mRNA expression)
5
4
3
2
1
0
###
***
SHAM
BDL
SHAM
BDL
N-RAS+/+
N-RAS-/-
C
Collagen III (mRNA expression)
30
20
10
0
#
SHAM
BDL
SHAM
BDL
N-RAS+/+
N-RAS-/-
D
αSMA (mRNA expression)
6
4
2
0
#
***
SHAM
BDL
SHAM
BDL
N-RAS+/+
N-RAS-/-
E
TIMP1 (mRNA expression)
100
80
60
40
20
0
#
***
***
SHAM
BDL
SHAM
BDL
N-RAS+/+
N-RAS-/-
F
IL1β (mRNA expression)
10
8
6
4
2
0
***
***
**
SHAM
BDL
SHAM
BDL
N-RAS+/+
N-RAS-/-

Suppl. Fig. 6

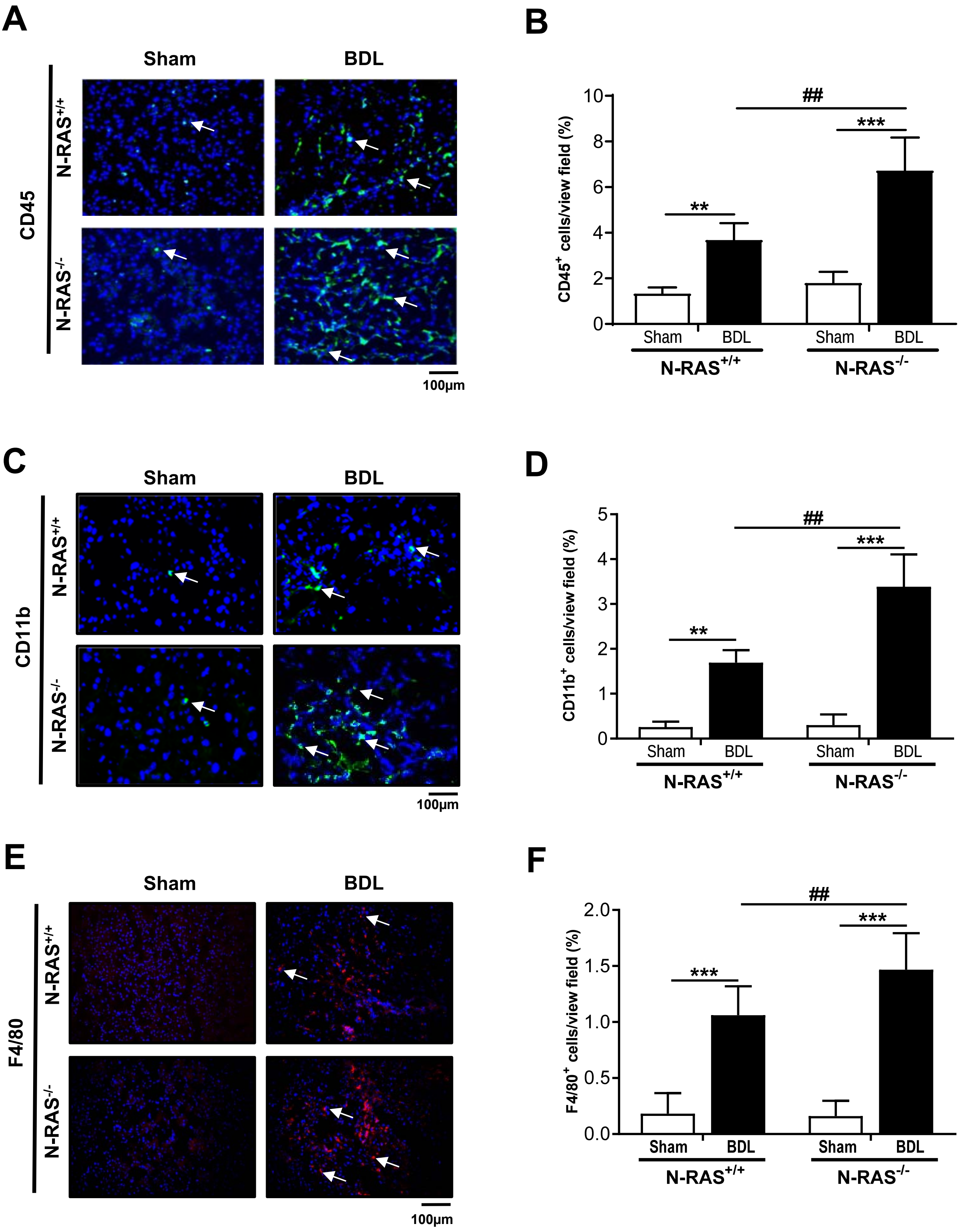
A
Sham
BDL
CD45
N-RAS+/+
N-RAS-/-
100μm
B
CD45+ cells/view field (%)
10
8
6
4
2
0
##
***
**
Sham
BDL
Sham
BDL
N-RAS+/+
N-RAS-/-
C
Sham
BDL
CD11b
N-RAS+/+
N-RAS-/-
100μm
D
CD11b+ cells/view field (%)
5
4
3
2
1
0
##
***
**
Sham
BDL
Sham
BDL
N-RAS+/+
N-RAS-/-
E
Sham
BDL
F4/80
N-RAS+/+
N-RAS-/-
100μm
F
F4/80+ cells/view field (%)
2.0
1.5
1.0
0.5
0.0
##
***
***
Sham
BDL
Sham
BDL
N-RAS+/+
N-RAS-/-

Suppl. Fig. 7

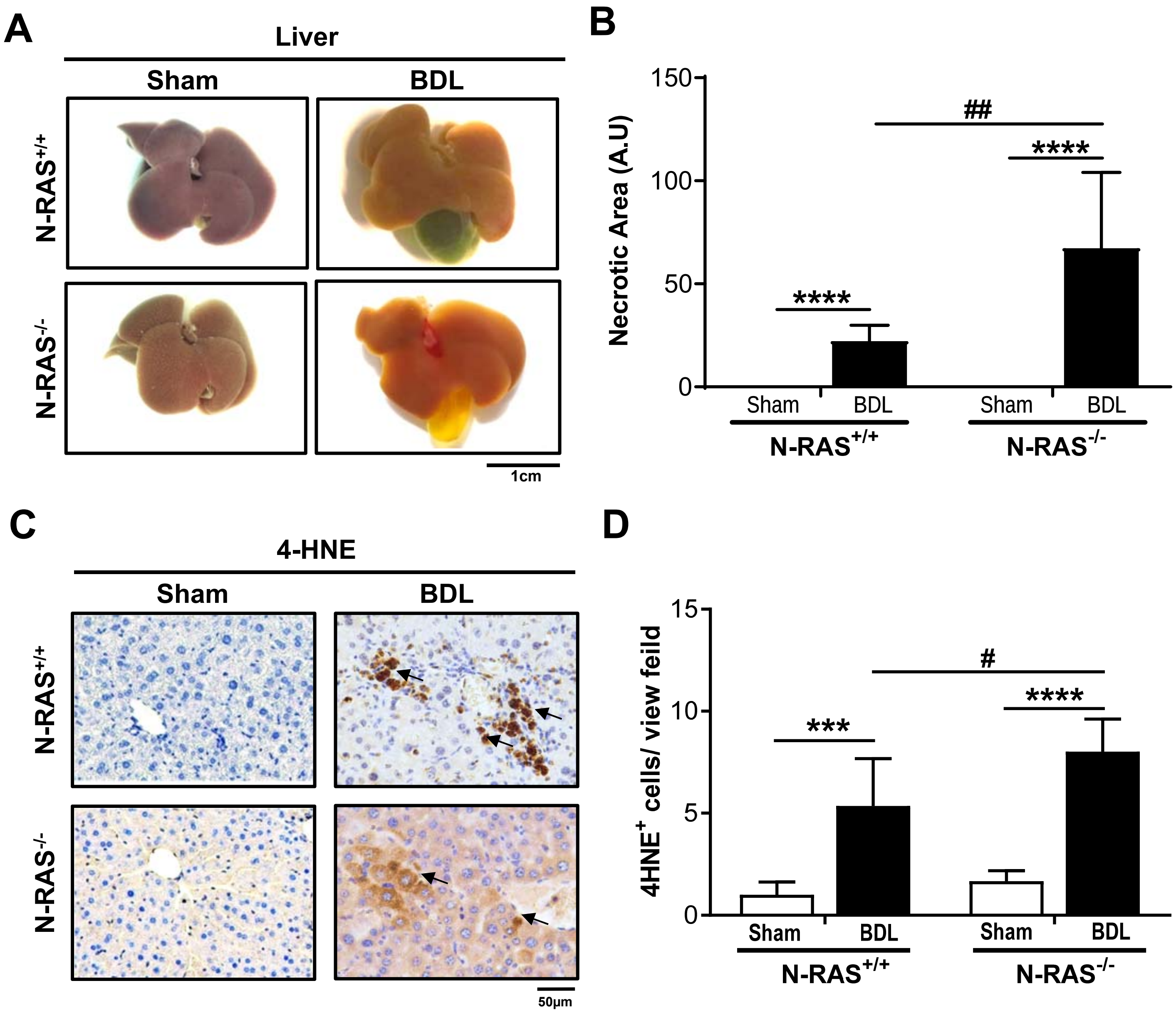
A
Liver
Sham
BDL
N-RAS+/+
N-RAS-/-
1cm
B
Necrotic Area (A.U)
150
100
50
0
##
****
****
Sham
BDL
Sham
BDL
N-RAS+/+
N-RAS-/-
C
4-HNE
Sham
BDL
N-RAS+/+
N-RAS-/-
50µm
D
4HNE+ cells/ view feild
15
10
5
0
#
***
****
Sham
BDL
Sham
BDL
N-RAS+/+
N-RAS-/-

Suppl. Fig. 8

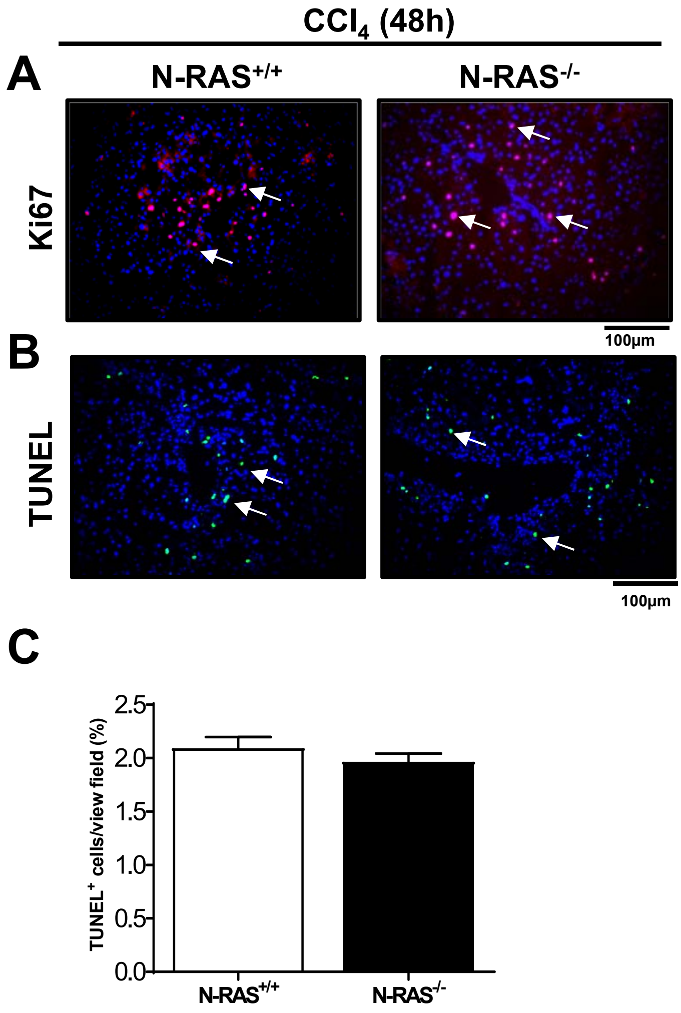

CCl4 (48h)
A
N-RAS+/+
N-RAS-/-
Ki67
100µm
B
TUNEL
100µm
C
TUNEL+ cells/view field (%)
2.5
2.0
1.5
1.0
0.5
0.0
N-RAS+/+
N-RAS-/-

Suppl. Fig. 9

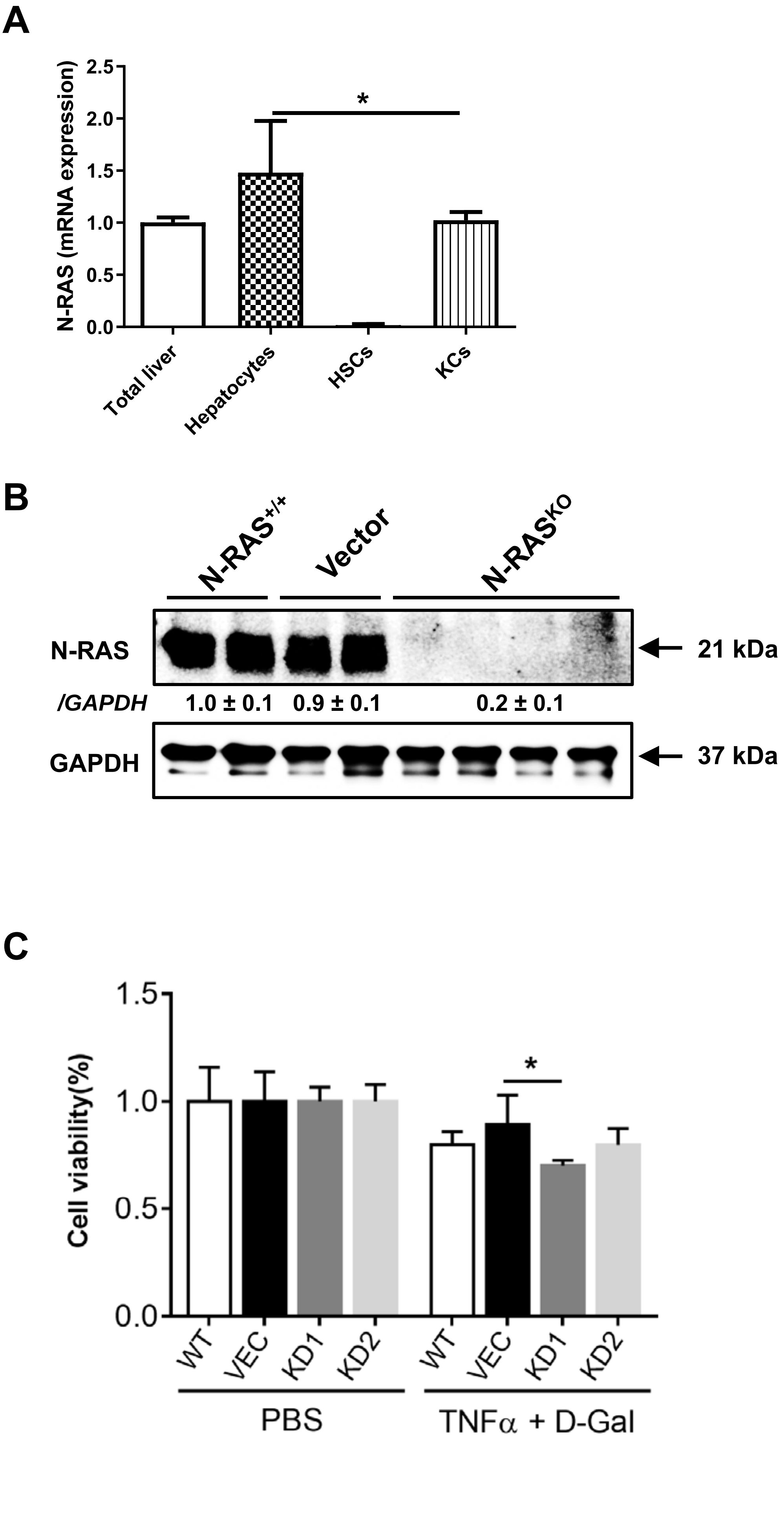
A
N-RAS (mRNA expression)
2.5
2.0
1.5
1.0
0.5
0.0
*
Total liver
Hepatocytes
HSCs
KCs
B
N-RAS+/+
Vector
N-RASKO
N-RAS
21 kDa
/GAPDH
1.0 ± 0.1
0.9 ± 0.1
0.2 ± 0.1
GAPDH
37 kDa
C
Cell viability(%)
1.5
1.0
0.5
0.0
*
WT
VEC
KD1
KD2
WT
VEC
KD1
KD2
PBS
TNFα + D-Gal

Suppl. Fig. 10

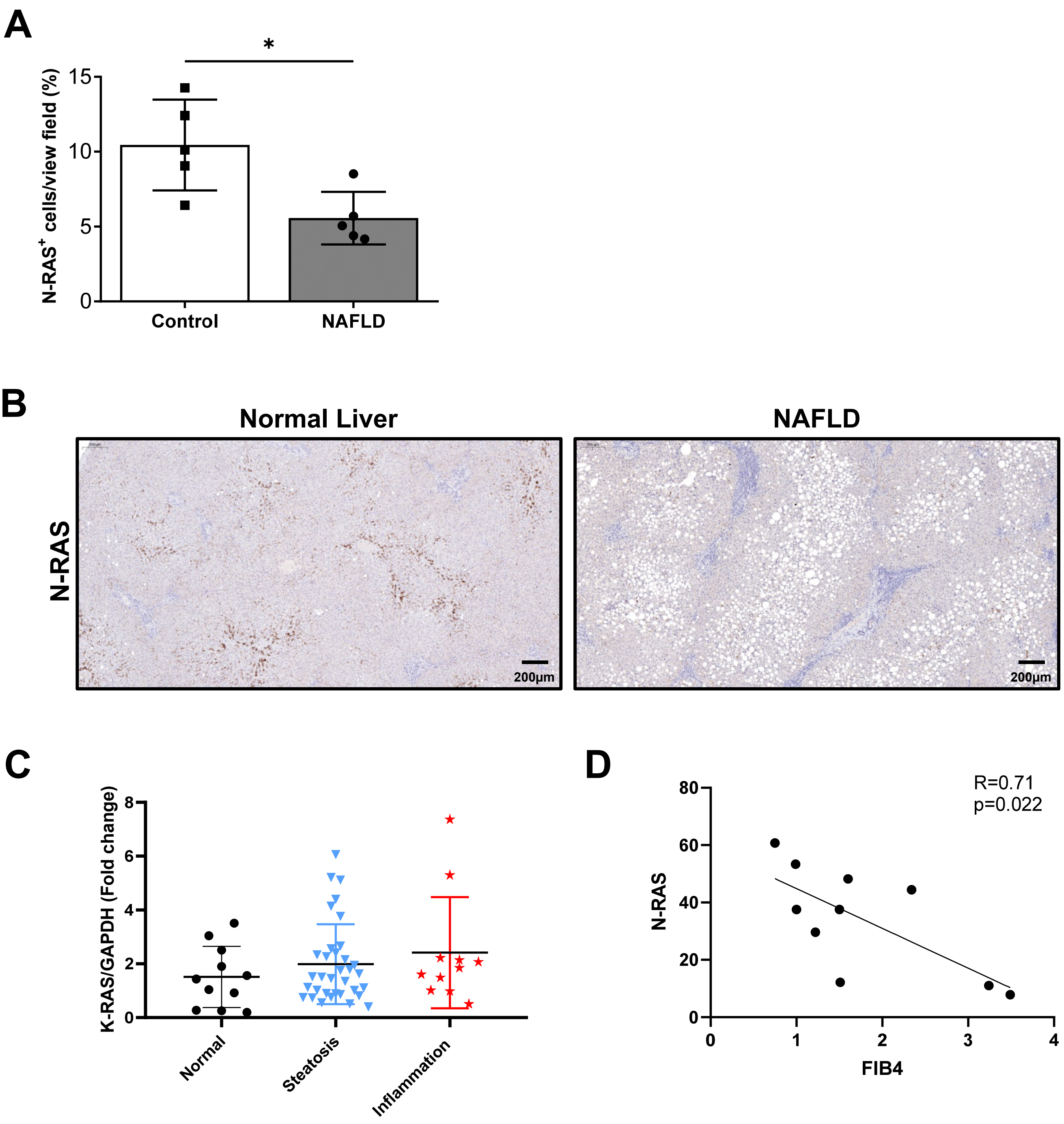

A
*
N-RAS+ cells/view field (%)
15
10
5
0
Control
NAFLD
B
Normal Liver
NAFLD
N-RAS
200µm
200µm
C
K-RAS/GAPDH (Fold change)
8
6
4
2
0
Normal
Steatosis
Inflammation
D
R=0.71
p=0.022
N-RAS
80
60
40
20
0
0
1
2
3
4
FIB4

Suppl. Fig. 11
