# Supplementary material for: Neuroblastoma RAS viral oncogene homolog (N-RAS) deficiency aggravates liver injury and fibrosis
Source: Cell Death Dis. 2023 Aug 10;14(8):514. doi: 10.1038/s41419-023-06029-y (PMC10415403; doi:10.1038/s41419-023-06029-y)
Supplement: Supplementary file 2 — Suppl. File uncropped WBs [file 41419_2023_6029_MOESM2_ESM.pptx]

## Slide 1
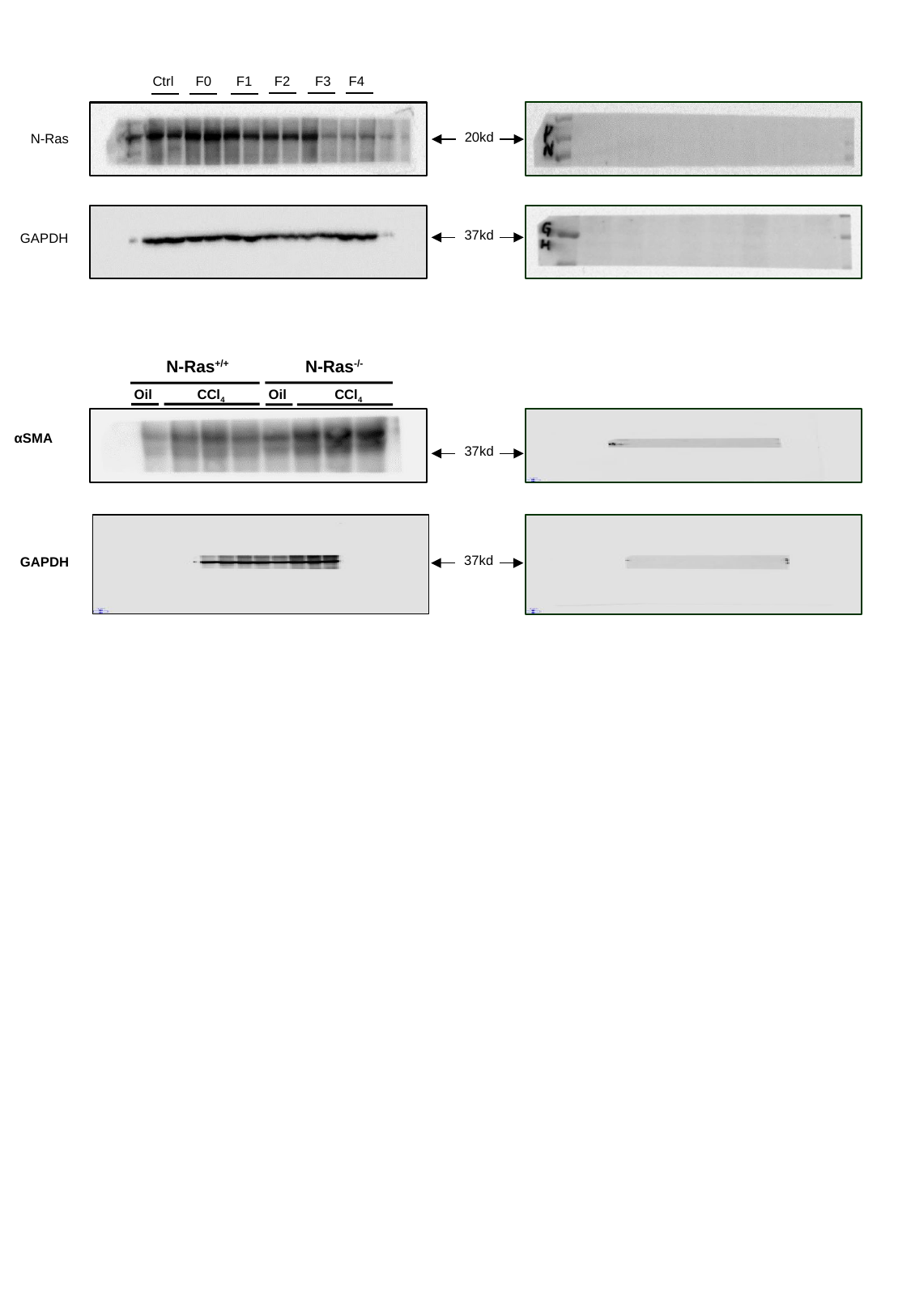

Ctrl
F0
F1
F2
F3
F4
20kd
N-Ras
37kd
GAPDH
N-Ras+/+
N-Ras-/-
Oil
CCl4
Oil
CCl4
αSMA
37kd
37kd
GAPDH

## Slide 2
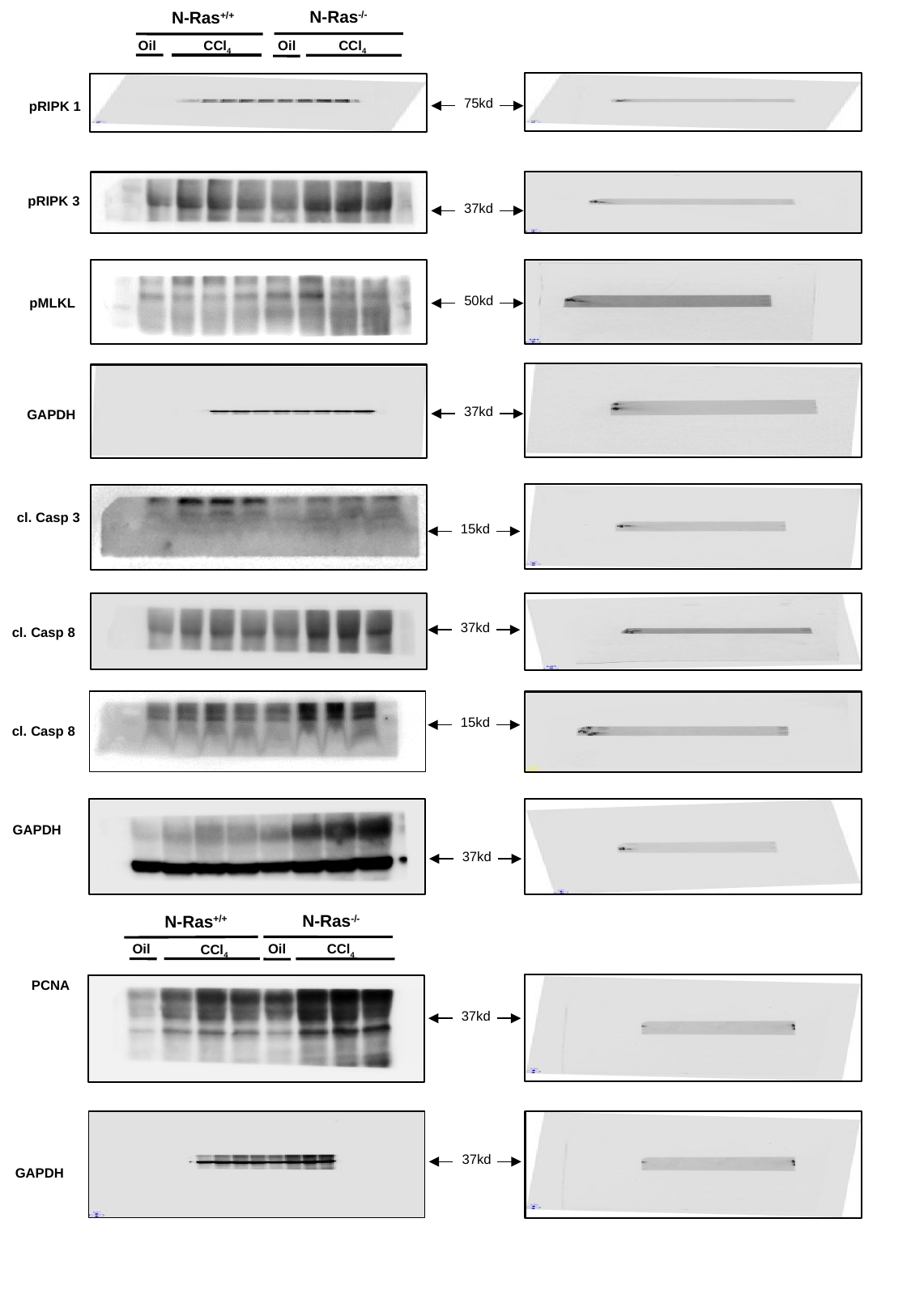

N-Ras-/-
N-Ras+/+
CCl4
 Oil
Oil
CCl4
75kd
pRIPK 1
pRIPK 3
37kd
50kd
pMLKL
37kd
GAPDH
cl. Casp 3
15kd
37kd
cl. Casp 8
15kd
cl. Casp 8
GAPDH
37kd
N-Ras-/-
N-Ras+/+
 Oil
Oil
CCl4
CCl4
PCNA
37kd
1.0 2.0 ± 0.2 1.9 3.8 ± 0.1 *
37kd
GAPDH

## Slide 3
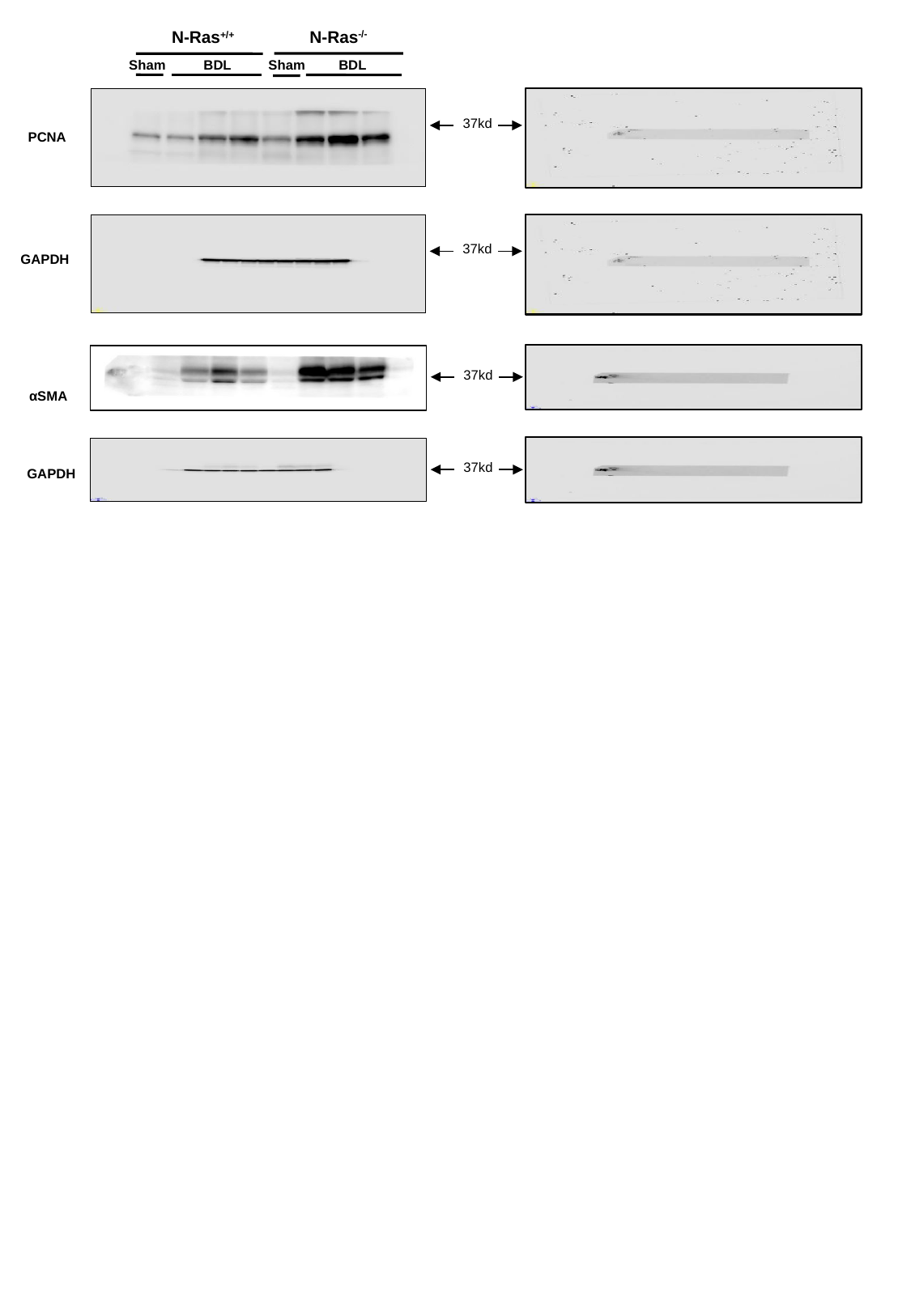

N-Ras-/-
N-Ras+/+
BDL
 Sham
Sham
BDL
37kd
PCNA
37kd
GAPDH
37kd
αSMA
37kd
GAPDH

## Slide 4
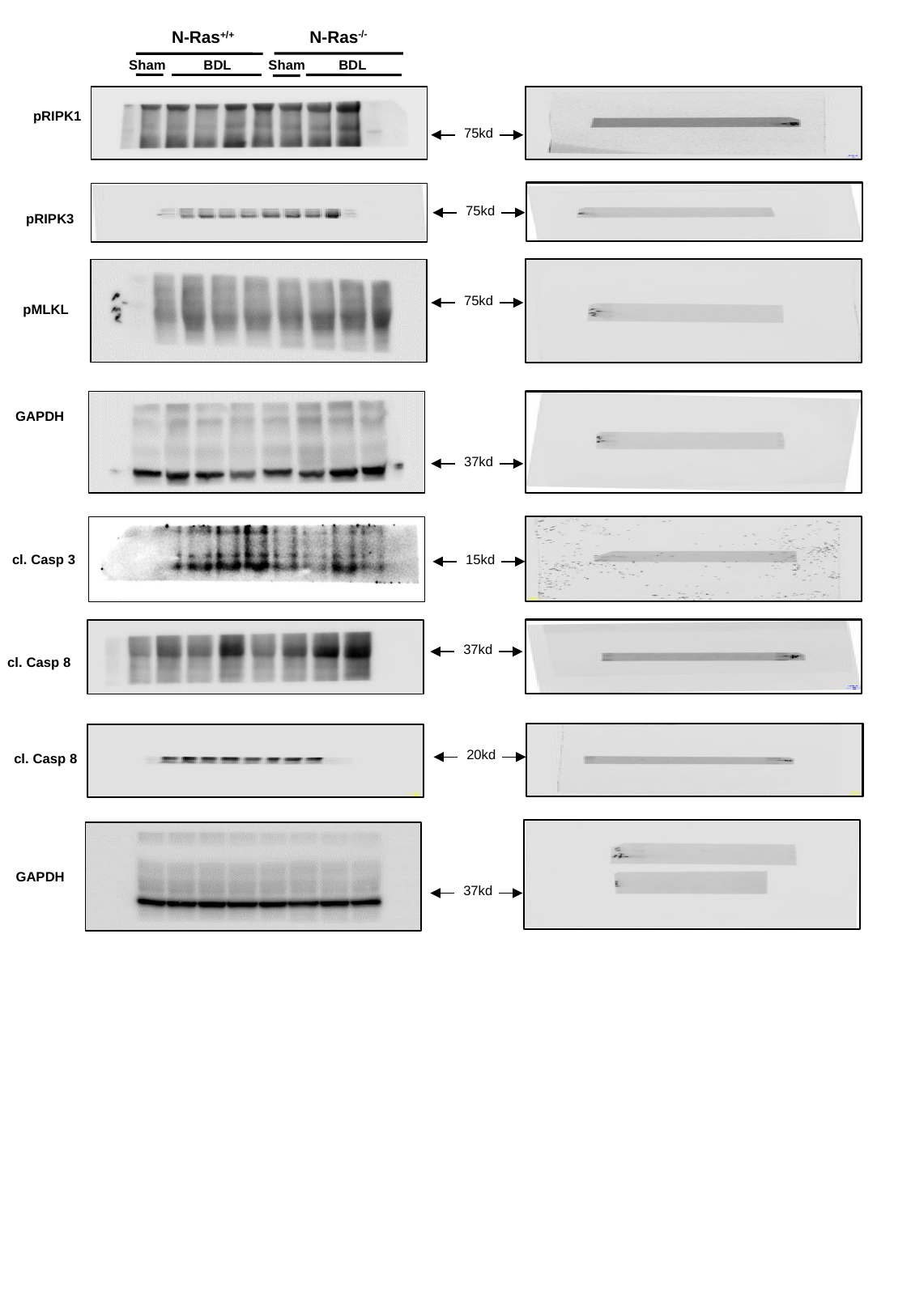

N-Ras-/-
N-Ras+/+
BDL
 Sham
Sham
BDL
pRIPK1
75kd
75kd
pRIPK3
75kd
pMLKL
GAPDH
37kd
15kd
cl. Casp 3
37kd
cl. Casp 8
20kd
cl. Casp 8
GAPDH
37kd

## Slide 5
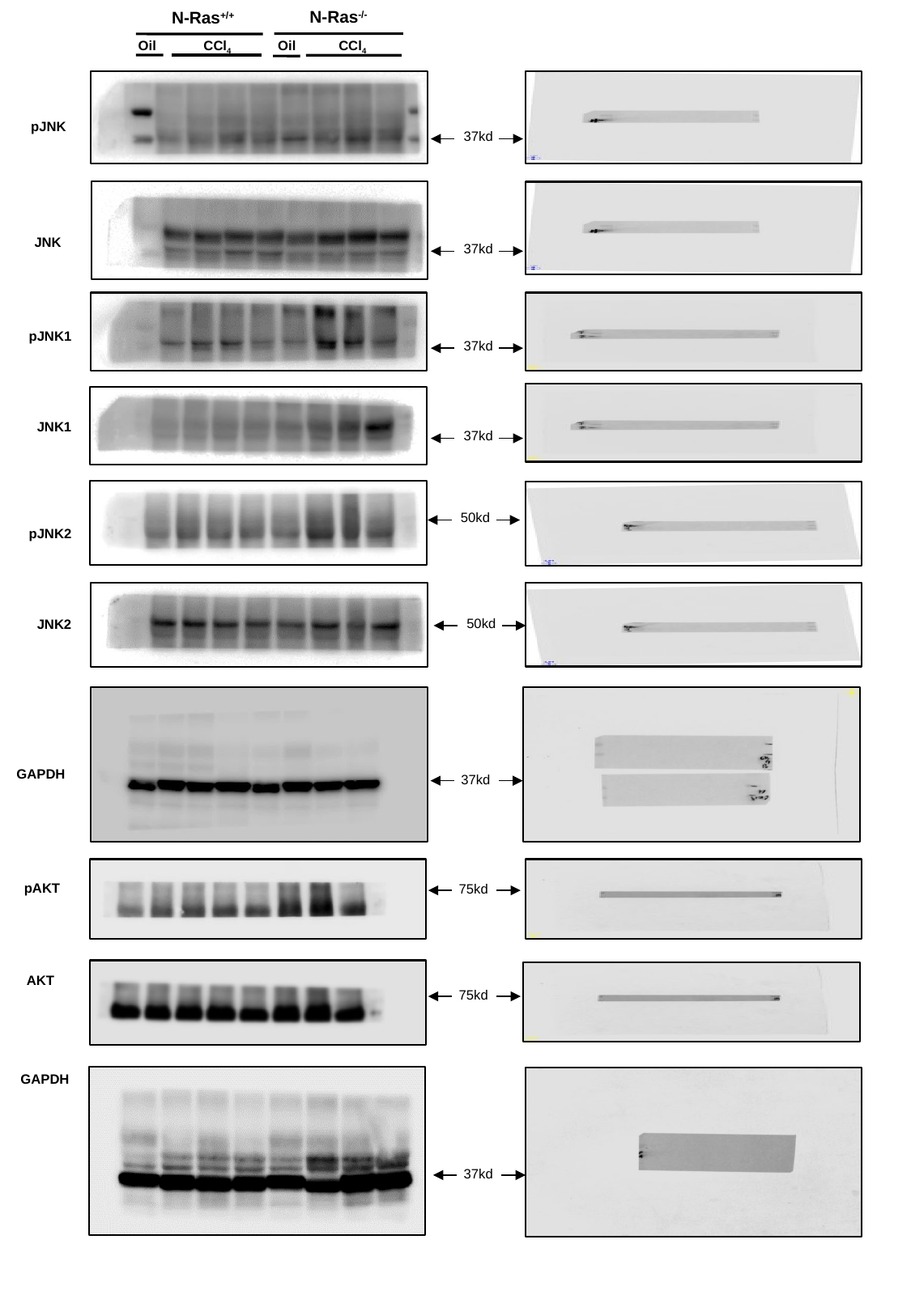

N-Ras-/-
N-Ras+/+
CCl4
 Oil
Oil
CCl4
pJNK
37kd
JNK
37kd
pJNK1
37kd
JNK1
37kd
50kd
pJNK2
50kd
JNK2
GAPDH
37kd
pAKT
75kd
AKT
75kd
GAPDH
37kd

## Slide 6
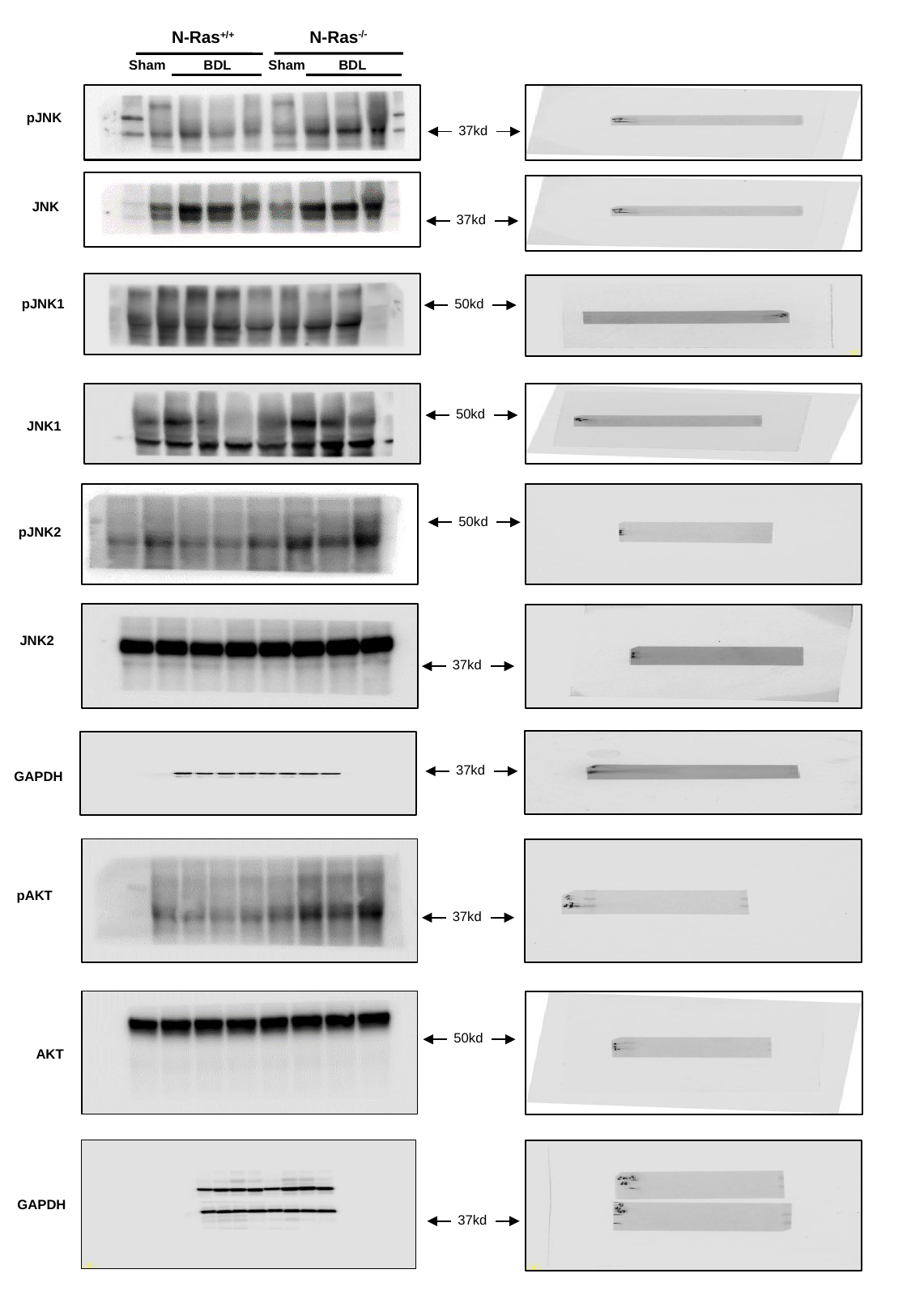

N-Ras-/-
N-Ras+/+
BDL
 Sham
Sham
BDL
pJNK
37kd
JNK
37kd
pJNK1
50kd
50kd
JNK1
50kd
pJNK2
JNK2
37kd
37kd
GAPDH
pAKT
37kd
50kd
AKT
GAPDH
37kd

## Slide 7
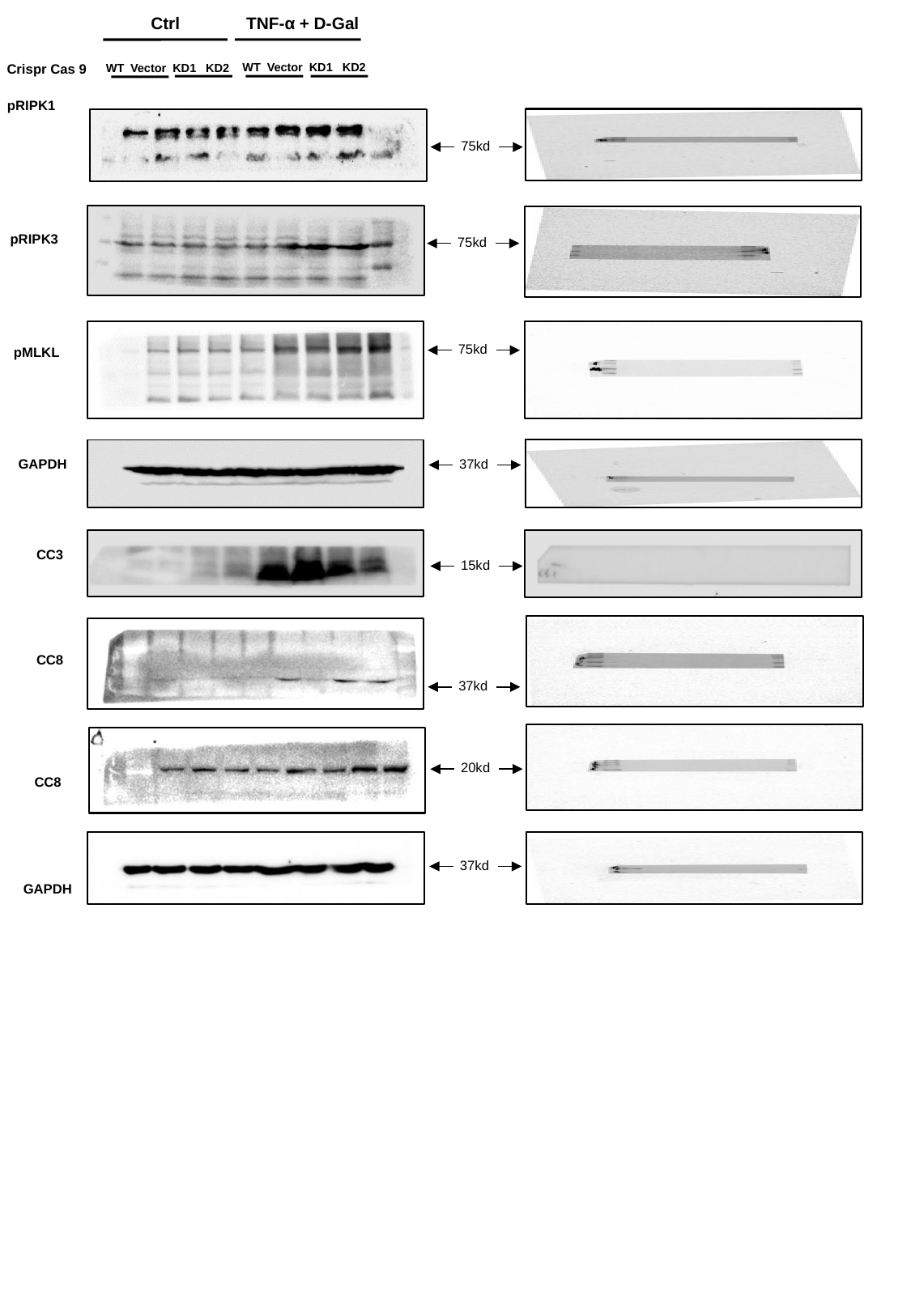

Ctrl
TNF-α + D-Gal
 WT Vector KD1 KD2
 WT Vector KD1 KD2
Crispr Cas 9
pRIPK1
75kd
pRIPK3
75kd
75kd
pMLKL
GAPDH
37kd
CC3
15kd
CC8
37kd
20kd
CC8
37kd
GAPDH

## Slide 8
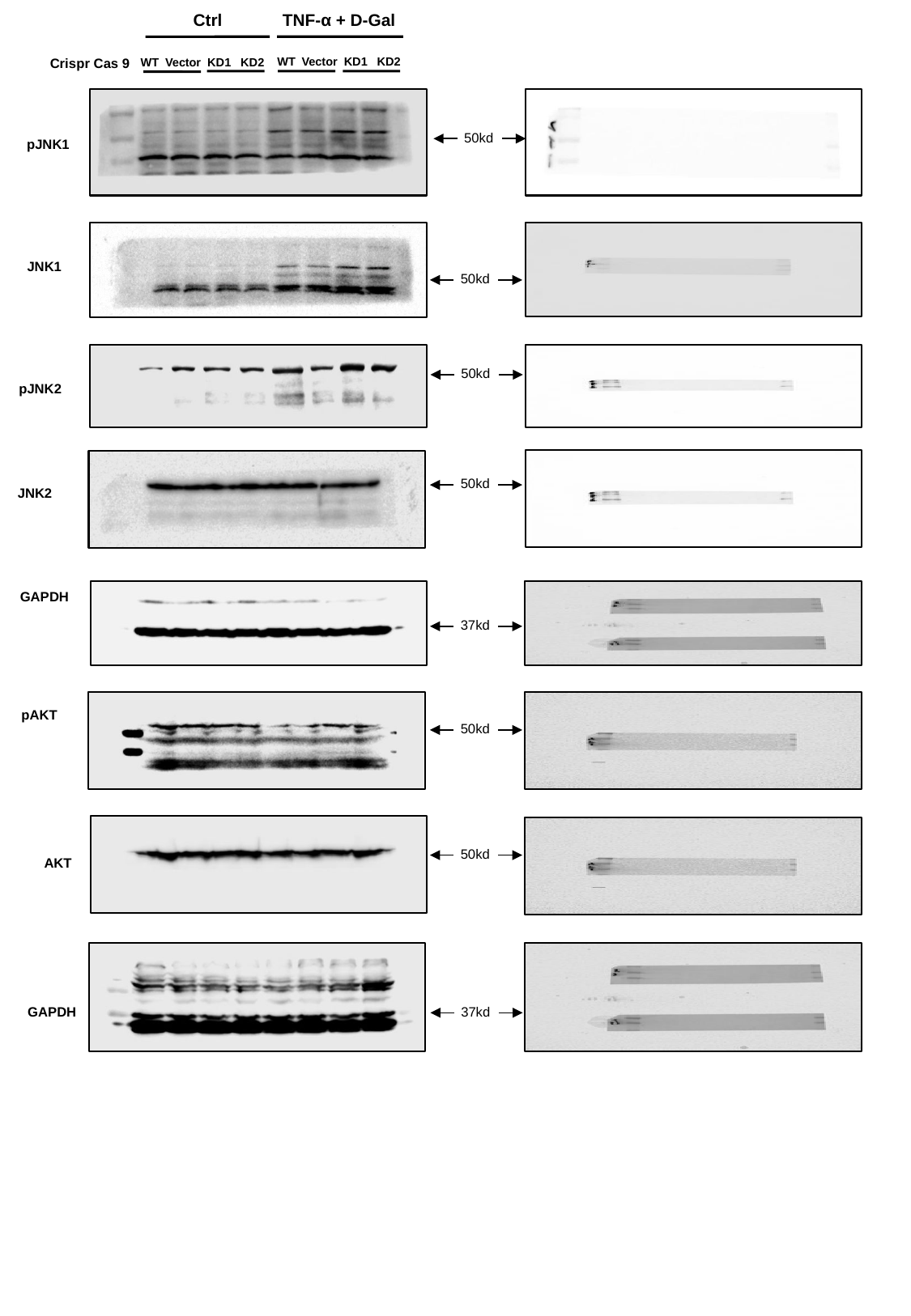

Ctrl
TNF-α + D-Gal
 WT Vector KD1 KD2
 WT Vector KD1 KD2
Crispr Cas 9
50kd
pJNK1
JNK1
50kd
50kd
pJNK2
50kd
JNK2
GAPDH
37kd
pAKT
50kd
50kd
AKT
GAPDH
37kd

## Slide 9
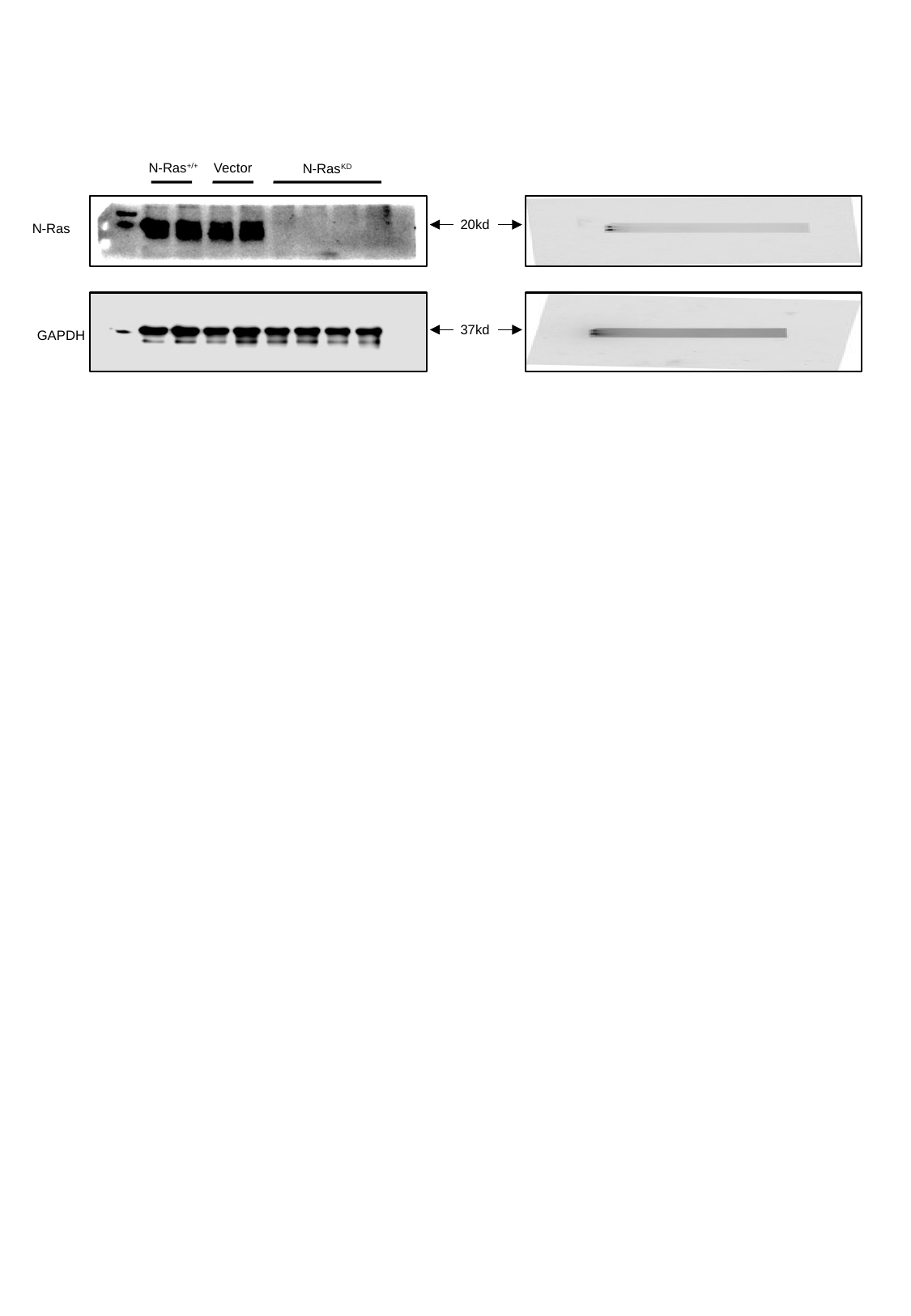

Vector
N-Ras+/+
N-RasKD
20kd
N-Ras
37kd
GAPDH
